# The Quasi-Coarse-Grained Dynamics Method to Unravel the Mesoscale Evolution of Defects/Damage during Shock Loading and Spall Failure of Polycrystalline Al Microstructures

**DOI:** 10.1038/s41598-017-12340-4

**Published:** 2017-09-28

**Authors:** Garvit Agarwal, Ramakrishna R. Valisetty, Raju R. Namburu, Arunachalam M. Rajendran, Avinash M. Dongare

**Affiliations:** 1 Department of Materials Science and Engineering and Institute of Materials Science, University of Connecticut, Storrs, Connecticut 06269 USA; 2 Computational and Information Sciences Directorate, U.S. Army Research Laboratory, Aberdeen Proving Ground, Maryland, 21005 USA; 3 Department of Mechanical Engineering, University of Mississippi, 201-B Carrier Hall, University, MS 38677 USA

## Abstract

A long-standing problem in modeling of shock response of metals is the ability to model defect nucleation and evolution mechanisms during plastic deformation and failure at the mesoscales. This paper demonstrates the capability of the “quasi-coarse-grained dynamics” (QCGD) simulation method to unravel microstructural evolution of polycrystalline Al microstructures at the mesoscales. The various QCGD simulations discussed here investigate the shock response of Al microstructures comprising of grain sizes ranging from 50 nm to 3.20 µm and correspond to system sizes ranging from 150 nm to 9.6 µm, respectively. The QCGD simulations are validated by demonstrating the capability to retain atomistic characteristics of the wave propagation behavior, plastic deformation mechanisms (dislocation nucleation, dissociation/recombination behavior, dislocation interactions/reactions), evolution of damage (voids), and evolution of temperature during shock loading. The capability to unravel the mesoscale evolution of microstructure is demonstrated by investigating the effect of grain size, shock pulse and system size on the shock response and spall failure of the metal. The computed values of spall strengths predicted using the QCGD simulations agree very well with the trend predicted by MD simulations and a strain rate dependence of the spall strength is proposed that fits the experimentally available values in the literature.

## Introduction

A critical challenge in the design of damage tolerant structural materials has been the ability to predict the materials response in extreme environments of shock loading. The response of metals is determined by evolution of defects (dislocations) in the microstructure during shock compression and their interactions under tri-axial tensile stresses to nucleate damage (voids)^1,2^. The mode of failure (spallation) requires an understanding of the atomistic characteristics related to nucleation and evolution of various types of dislocations that create weak regions to serve as nucleation sites for voids in deformed microstructures. The spall strength, defined as “*the peak tensile pressure required to nucleate voids”*, is used to quantify damage tolerance of metals under shock loading conditions and is observed to vary with loading strain rates and microstructure of the metal^1,2^. For the case of Al microstructures, this value of spall strength has been investigated in several studies using plate impact experiments^2–8^ for strain rates of ~10^5^ s^−1^ to ~10^7^ s^−1^ and using laser shock experiments^9–13^ for higher strain rates of ~10^7^ s^−1^ up to ~10^10^ s^−1^. However, the role of microstructure and loading conditions (shock compression pressures, and temperature) is still unclear. This limited understanding is attributed to experimental challenges to characterize/quantify the evolution of defects at the short lengths (nanometers to microns) and times of the phenomena (picoseconds to microseconds).

As a result, several computational methods^14–21^ have been developed that use phenomenological and constitutive relationships to account for the microstructural response of a metal under shock loading conditions. A few recently developed successful thermoelastic-viscoplastic models^14,22–24^ are also able to model the shock response by reproducing experimentally observed decay of the elastic precursor in single crystal and polycrystalline Al samples^25–27^. These models, however, have to make several approximations^14,24^ for plastic deformation processes that are based on nucleation of mobile and immobile dislocations, and dislocation multiplication/annihilation mechanisms based on mean velocities of dislocations as measured experimentally^28^. However, the limited capability of experiments to quantify these mechanisms at high strain rates (10^5^ s^−1^ and larger), poses a challenge for the development of such models. Classical molecular dynamics (MD) simulations can provide critical insights in the atomic scale mechanisms of these phenomena and complement experimental analysis as well as continuum simulations. Successful examples of MD simulations have investigated mechanisms related to nucleation and evolution of defects^29–35^ during spall failure. Such insights enable the development of theoretical models for dependence of the spall strengths on the grain size of metals^36^. The MD simulations, however, due to their time and length scale capabilities, are limited to system sizes that are up to a few hundred nanometers and result in strain rates of ~10^10^ s^−1^ under shock loading conditions using reasonable computing resources.

While it can be argued that access to and growth of the current high performance computing resources allows MD simulations on the order of a few Billion atoms, these simulations enable modeling of these phenomena at strain rates of 10^9^ s^−1^ under shock loading conditions. Such simulations, however, then present the challenge of ‘Big Data’ that is generated (tens to hundreds of Terrabytes for each simulation) that needs to be analyzed (post-processing), visualized and stored. As a result, the capability to run several such simulations to investigate the shock response and unravel the evolution of microstructure is largely limited. A critical gap, therefore, exists in the modeling of the shock response of the metals between the atomic scales and the experimental scales. The recently developed “quasi-coarse-grained dynamics” (QCGD) method^37^ scales up the MD simulations to model the mesoscale behavior by coarse-graining the atomistic microstructure using representative atoms (R-atoms) and using scaled interatomic potentials. The QCGD framework, as demonstrated for single crystal systems, is able to reproduce the MD predicted shock deformation and spall failure of FCC metals^37^.

This paper demonstrates the capability of the QCGD method to retain the MD predicted evolution of defects and temperature in the metal microstructures as well as the spall failure behavior and spall strength values for nanocrystalline Al systems. In addition, this paper demonstrates the capability of the QCGD simulations to model atomistic characteristics related to nucleation, dissociation and recombination mechanisms of dislocations, as well as the dislocation reactions in polycrystalline Al systems. The polycrystalline Al systems correspond to grain sizes ranging from 50 nm to 3.20 µm that correspond to system sizes ranging from 150 nm to 9.60 µm, respectively using the higher levels of coarsening. The QCGD simulations are able to unravel the evolution of microstructure (defects/damage) at the mesoscales. The QCGD prediction of the shock wave propagation, dislocation density evolution, temperatures, and the damage nucleation and evolution behavior in these polycrystalline Al microstructures is beyond the current capability of MD simulations and allows to investigate the role of microstructure and loading conditions on the shock response and spall failure of Al systems. As will be discussed later, QCGD simulations can provide atomistic information related to dislocation densities and spall strengths and are able to bridge the mesoscale gap and complement the experimental studies.

The QCGD simulations retain the atomistic characteristics related to nucleation, evolution, and interaction of dislocation structures as predicted by the MD simulations. This capability is incorporated through modeling the collective evolution of defects rather than modeling individual defects using representative defect structures (discussed in the Supplemental Information: S2–S3). For example, the QCGD framework uses R-atoms to describe the collective dynamics of several atoms in the atomistic microstructure. Such a framework requires scaled interactions between the R-atoms to retain the atomic scale energetics as predicted in the atomic scale microstructure in the bulk, grain boundaries, surfaces, etc. Similarly, a point defect in the QCGD framework represents several point defects in an atomic scale microstructure and a dislocation (and a fault) in the QCGD framework represents several dislocations (faults) in an atomic scale microstructure. Such a representation of defects, however, scales the energies required to nucleate a defect in the QCGD simulation as compared to that in the MD simulation. For example, the L2-QCGD simulations will nucleate one stacking fault when the system has energy that corresponds to nucleation of two atomistic stacking faults. As a result, the stacking fault energy and the width of the stacking fault for a L2-QCGD simulation is twice that predicted for an MD simulation. This scaling of the nucleation energetics, results in a slight strengthening of the system as compared to the atomistic system. Similarly, the L4-QCGD, L8-QCGD and L16-QCGD simulations will nucleate one stacking fault when the system has enough energy that corresponds to nucleation of four, eight, and sixteen atomistic stacking faults, respectively. The stacking fault energy and the width of the stacking fault for the L4-QCGD, L8-QCGD and L16-QCGD simulations is four times, eight times and sixteen times that predicted for an MD simulation. The applicability/validity of these scaling relationships for various levels of coarsening is determined by the microstructure under study and hence limits the level of coarsening that can be used to model a microstructure. The microstructural aspects (grain size) need to be large enough to make such representations of defects in the QCGD simulations.

**Figure 1:**
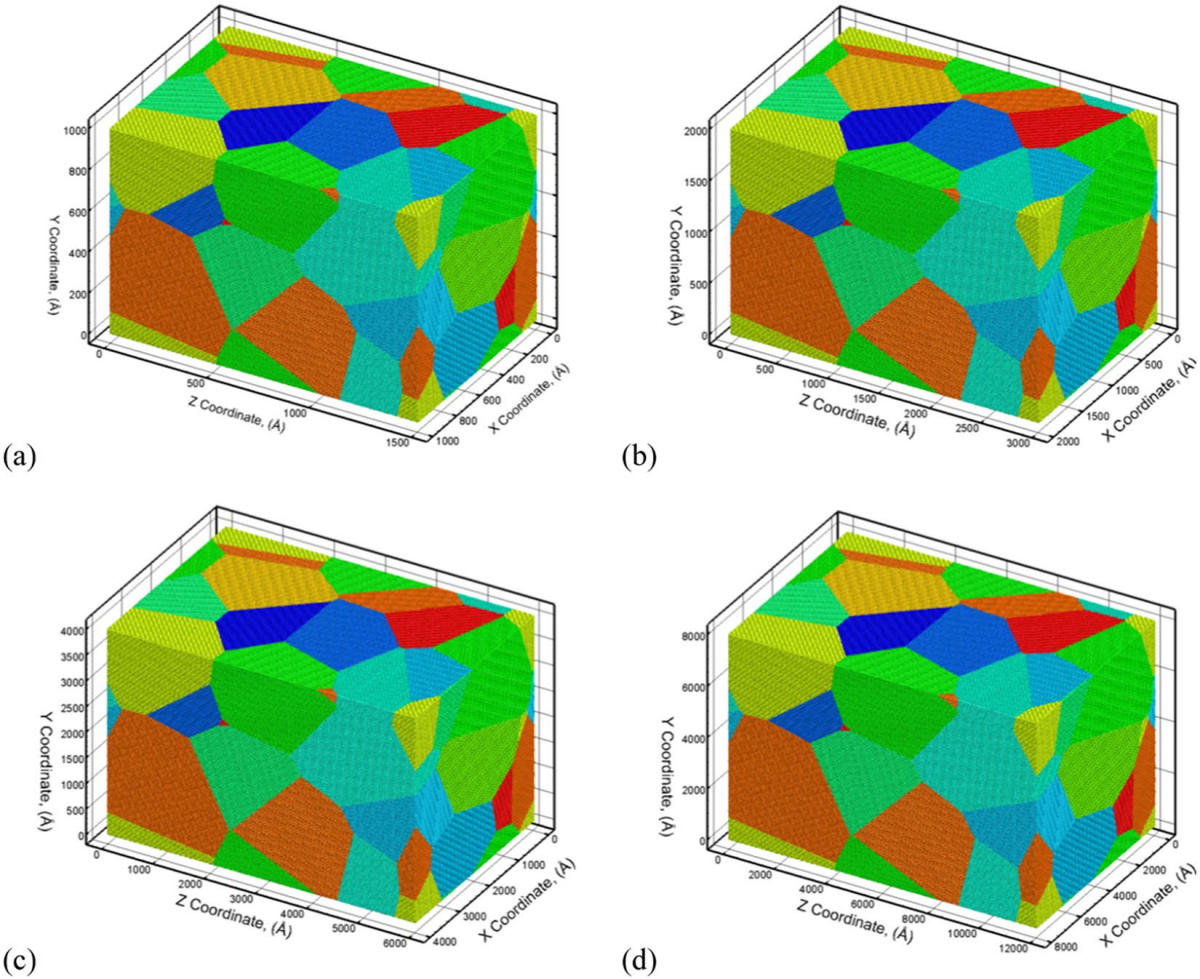
The validation for the L2-QCGD simulation is based on the predictions of the MD simulations of a 50 nm grain sized 100 nm × 100 nm × 150 nm polycrystalline Al system as shown in (**a**). Similarly, the validation of the L4-QCGD simulations is based on the L2-QCGD simulations of a 100 nm grain sized 200 nm × 200 nm × 300 nm polycrystalline Al system as shown in (**b**), the validation of the L8-QCGD simulations is based on the L4-QCGD simulations using a 200 nm grain sized 400 nm × 400 nm × 600 nm polycrystalline Al system as shown in (**c**), and the validation of the L16-QCGD simulations is based on the L8-QCGD simulations for a 400 nm grain sized 800 nm × 800 nm × 1.2 µm polycrystalline Al system as shown in (**d**). A grain identity number is used to color the atoms to indicate that the orientation relationships and the numbers of grains are the same in all the microstructures.

As a result, polycrystalline Al microstructures are used to validate the capability of various levels of the coarsening in QCGD simulations to model the shock response (shock wave velocities, shock pressures, dislocation density evolution, etc.) and spall failure behavior (void nucleation, spall strengths, etc.). The initial polycrystalline structures with grain sizes of 50 nm, 100 nm, 200 nm and 400 nm (corresponding to system sizes of 150 nm, 300 nm, 600 nm to 1.2 microns) used to validate L2, L4, L8, and L16 levels, respectively, are shown in Fig. 1 with atoms/R-atoms colored according to an identity of each grain. The various polycrystalline Al samples are created using the same number of R-atoms and the same grain orientation relationships for all the systems. The initial polycrystalline microstructures comprise of pre-existing dislocations generated due to the misorientation at the boundaries^34^. The pre-existing dislocation density for the various types of dislocations in the initial systems created for the various levels of coarsening are provided in Supplemental Information S4 and are tabulated in Table S4. It can be seen that while the coarsening of the microstructure does not retain the exact dislocation densities, the various levels of coarsening retain the relative fractions of the various types of pre-existing dislocations at the grain boundaries for the polycrystalline systems. The values of the dislocation density suggests that an ‘*atomic scaling factor*’ can be defined to predict a ‘*representative atomistic dislocation density*’ based on the values computed for the various levels of coarsening. For each of the grain sizes chosen for validation (50 nm, 100 nm, 200 m, and 400 nm), the predicted atomic level dislocation densities for the various types of pre-existing dislocations using the scaling factors are tabulated in Table 1. The predicted representative atomic level dislocation densities compare very well with the experimentally calculated values^38–41^.

**Table 1:** Calculated atomic level pre-existing dislocation densities for the various microstructures for the various levels of coarsening.

Dislocation	Perfect × 10^15^ (m^−2^)	Shockley × 10^15^ (m^−2^)	Hirth × 10^11^ (m^−2^)	Frank × 10^14^ (m^−2^)	Stair-rod × 10^12^ (m^−2^)	Total × 10^15^ (m^−2^)
*d* = 50 nm	22.09	12.93	45.0	14.7	10.0	35.18
*d* = 100 nm	6.20	3.29	4.60	4.77	2.61	9.97
*d* = 200 nm	3.77	1.74	0.43	2.48	0.52	5.75
*d* = 400 nm	3.56	1.28	0.07	1.98	0.96	5.039

The validation of scaling relationships is first carried out for the L2-scaling in QCGD simulations based on the prediction of the shock wave velocities, shock pressures, defect evolution, temperature evolution and spall strength for a 50 nm grain sized 100 nm × 100 nm × 150 nm polycrystalline Al system using ~11 Million R-atoms and the same grain orientation relationships using MD simulations (~89 Million atoms). The L2-QCGD simulations are then used to validate the L4-QCGD simulations based on the prediction for a 100 nm grain sized 200 nm × 200 nm × 300 nm polycrystalline Al system (corresponds to an atomistic system of ~712 Million atoms) and the same grain orientation relationships. Similarly, the L4-QCGD simulations are then used to validate the L8-QCGD simulations using a 200 nm grain sized 400 nm × 400 nm × 600 nm polycrystalline Al system (atomistic system of ~5.7 Billion atoms) and the same grain orientation relationships, and the L16-QCGD simulations are validated using the results of L8-QCGD simulations for a 400 nm grain sized 800 nm × 800 nm × 1.2 µm polycrystalline Al system (atomistic system of ~45.6 Billion atoms) and the same grain orientation relationships. More details on the validation of the levels of coarsening (L2-scaling to L16-scaling) are provided in the Supplemental Information S5 to S8.

## Results and Discussions

**Figure 2:**
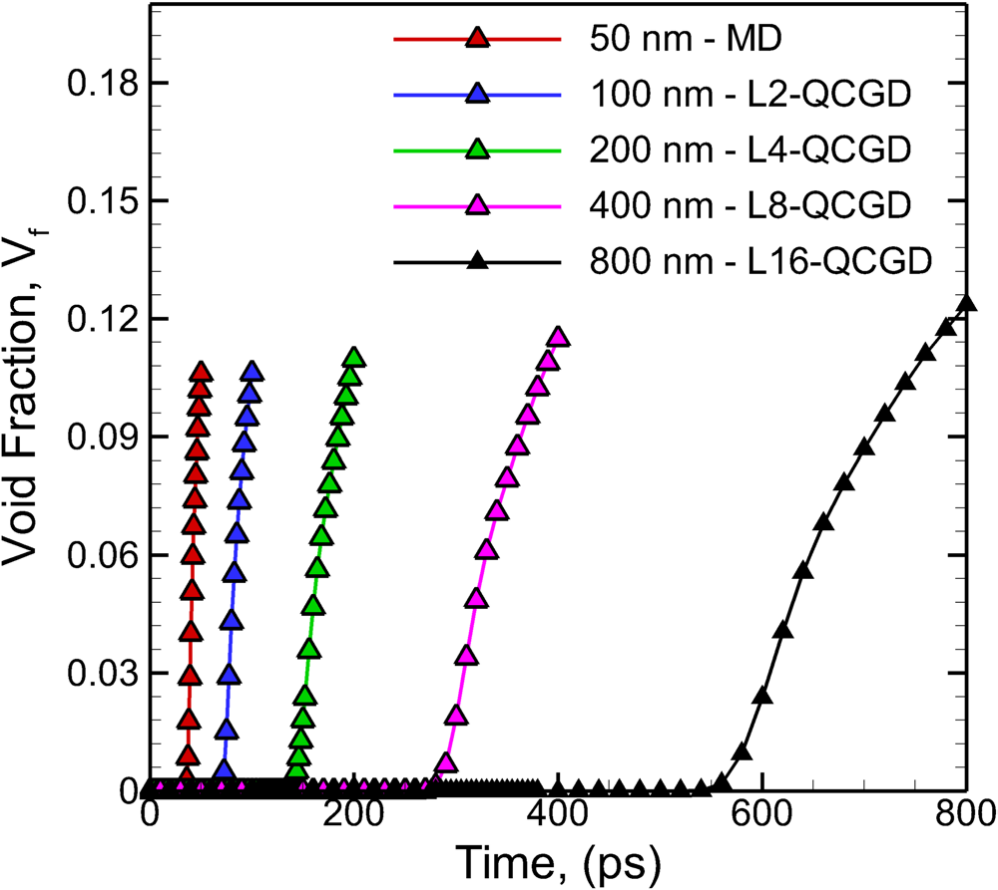
The variation of the void fraction as a function of time for the various simulations discussed in Supplemental Information S5 to S8 using the microstructures shown in Fig. 1.

These results discussed in the Supplemental Information S5 to S8 demonstrate the capability of QCGD simulations to investigate the spall behavior of polycrystalline Al systems. Since, the types of grain boundaries and the grain orientation relationships are the same for all the microstructures chosen, the response of these GBs to the shock are expected to be the same if experiencing the same loading conditions (shock pressures, strain rates, temperature, etc.) and the deviations in the response will be attributed to the grain size of the metal. The scaling of system size results in variations in strain rates of loading and hence results in variations in nucleation and evolution of voids during spall failure. A plot showing the variation of the void fraction (V_f_) for the various simulations discussed in the Supplemental Information S5 to S8 is shown in Fig. 2. More details about the nucleation and growth of voids in these simulations are provided in Supplemental Information S10. These simulations are also analyzed to compute the “*rate of growth and coalescence of voids*” and the computed rates are tabulated in Table 2. This variation of growth of voids computed here can be used to parameterize damage evolution models that include strain rate and microstructure dependence in continuum simulations.

**Table 2:** Calculated values of rate of evolution of void fraction for the various strain rate (s^−1^) using a velocity of 1 km/s and varying pulse durations for the piston for the various polycrystalline Al microstructures.

Grain Size (nm)	Strain Rate, $\dot{\varepsilon }$ (s^−1^)	Void Growth rate, ${\dot{V}}_{f}$ (ps^−1^)
50 (MD)	7.17 × 10^9^	0.0075
100 (L2)	3.46 × 10^9^	0.0034
200 (L4)	1.74 × 10^9^	0.0017
400 (L8)	8.17 × 10^8^	0.00089
800 (L16)	4.43 × 10^8^	0.00051
3200 (L64)	1.15 × 10^8^	0.00014

In addition, the scaling relationships have been validated to a level of coarsening of L32-scaling and L64-scaling for the QCGD simulations by reproducing the atomic scale characteristics of the shock wave propagation velocities, evolution of dislocation density fractions, the spall width, spall strengths and the evolution of temperature. Thus, the various levels of coarsening in the QCGD simulations (up to L64-scaling), now validated, can be used to investigate the role of grain size, pulse duration, system size, etc. on the mechanisms of evolution of defect densities and damage during shock loading and spall failure. A few example systems used here comprise of L16-QCGD simulations of an 800 nm grain sized polycrystalline Al system with the same grain orientation relationships. This system, with dimensions of 1.6 µm × 1.6 µm × 2.4 µm, comprises of ~89 Million R-atoms and represents an atomistic system of ~365 Billion atoms. The largest system modeled here is using the L64-QCGD simulations of a 3.2 µm grain sized system with dimensions 6.40 µm × 6.4 µm × 9.60 µm and corresponds to a size of ~89 Million R-atoms using L64-scaling and represents an atomistic system of ~24 Trillion atoms. These length scales enable the evaluation of the strain rate dependence of the spall strength for polycrystalline Al systems. These QCGD simulations demonstrate the capability to unravel the microstructural evolution in polycrystalline Al systems under conditions of shock loading and spall failure. A few example simulations are discussed below.

**Figure 3:**
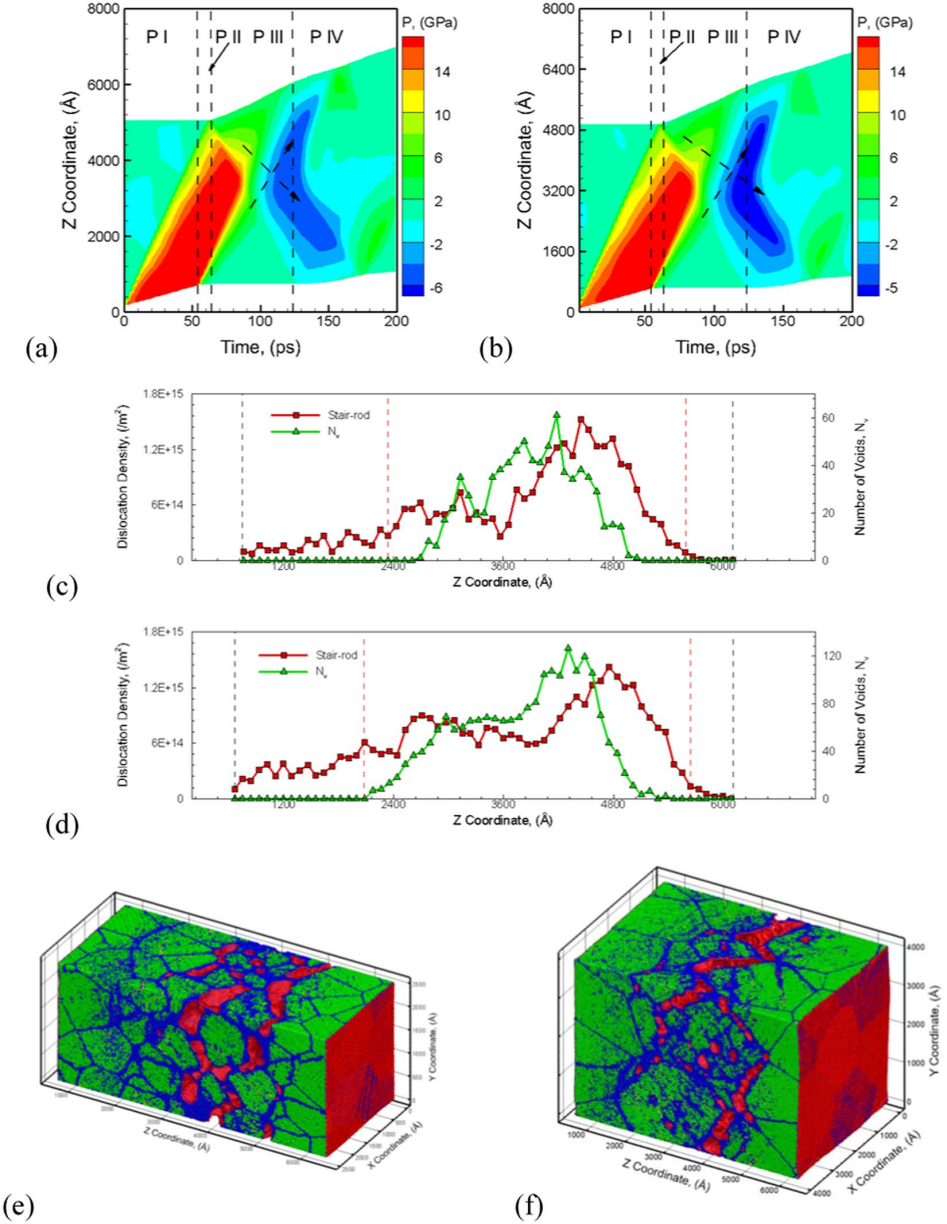
The plots for the evolution of pressure during spall failure predicted by L4-QCGD simulation for a velocity of 1 km/s for the piston and pulse duration of 50 ps of 100 nm and 200 nm grain sized polycrystalline Al system are shown in (**a**) and (**b**), respectively. The distribution of number of voids and density of stair-rod dislocations as a function of sample length in the shock direction for 100 nm and 200 nm grain sized polycrystalline Al system are shown in (**c**) and (**d**) respectively. The variations in the failure behavior is observed in the snapshots showing the microstructure in (**e**) for the 100 nm grain sized system and in (**f**) for the 200 nm grain sized system at a time of 154 ps. The coloring of the atoms is used to identify defects, surface and stacking sequences using a combination of CNA and CSP values. The FCC stacked atoms are colored green, HCP stacked atoms are colored yellow, surface atoms are colored red and the disordered atoms are colored blue.

The first set of simulations discussed here investigates the role of grain size on the defects and damage evolution in polycrystalline Al systems with L4-scaling relationships for the QCGD simulations. The first system created with L4-scaling relationships comprises of a 100 nm grain sized 0.25 µm × 0.25 µm × 0.50 µm system consisting of ~29 Million R-atoms (atomistic system of ~ 1.85 Billion atoms). The second system created with L4-scaling comprises of a 200 nm grain sized 0.40 µm × 0.40 µm × 0.50 µm system consisting of ~75 Million R-atoms (atomistic system of ~ 4.8 Billion atoms). The L4-QCGD simulations are carried out for an inward velocity (in the Z direction) of 1 km/s for the piston (bottom end of the system in Z direction) and pulse duration of 50 ps using a time-step of 16 fs. Figure 3(a) and (b) show the comparison of the temporal evolution of pressure for the 100 nm grain sized system and the 200 nm grain sized system along the length of the sample. These plots enable the investigation of the shock wave propagation (red indicates compressive pressure), reflection and interactions to generate a tri-axial tensile wave (blue indicates tensile pressure). The discussion of the wave propagation behavior can be focused on the four phases of wave propagation behavior: Phase I (P-I) spans the propagation of compression wave for the given pulse duration; Phase II (P-II) begins at the arrival of the tail of the compressive wave and ends when the compression wave reaches the rear surface; Phase III (P-III) begins at the expansion of the rear surface and ends when voids are nucleated; and Phase IV (P-IV) corresponds to the growth of nucleated voids as the tri-axial tensile wave travels towards the piston. Since the loading conditions and the dimensions of the two systems in the shock direction are the same, the spall region width is predicted to be similar for the two systems. A comparison of the evolution of voids fraction for the two simulations is shown in Figure S9(a). While the void fraction evolution is very similar for the two systems, the effect of microstructure can be investigated by understanding the evolution of stair-rods in the metal during nucleation of voids. A distribution of number of voids is plotted along with a distribution of stair-rods along the length of the sample in Fig. 3(c) and (d) at a time corresponding to peak number of voids in the metal for the two simulations discussed here (t = 129.6 ps for 100 nm grain sized system and t = 133.4 ps for the 200 nm grain-sized system). The distribution of voids in the material coincides with a high density of stair-rods. A variation of the density of Stair-rods and the number of voids as a function of time (as shown in Figure S9(b) and (c)) suggests that the formation of Stair-rods is a precursor for the nucleation of individual voids in the spall region and Stair-rods are likely to form nucleation sites for voids in the spall region. Given the same spall width, a 100 nm grain sized system has a high density of grain boundaries as compared to the 200 nm system and hence voids are observed to nucleate at the grain boundaries as shown in Fig. 3(e). For the case of the 200 nm system, a higher density of Stair-rods are formed due to larger grain interior regions and hence a larger number of nucleated voids. The larger grain interior region for 200 nm system creates a distribution of Stair-rods in grain interior regions in addition to that at grain boundaries and hence results in void nucleation at grain boundaries as well as the grain interior regions as shown in Fig. 3(f). This correlation between Stair-rods and number of voids is observed in all simulations discussed here.

**Figure 4:**
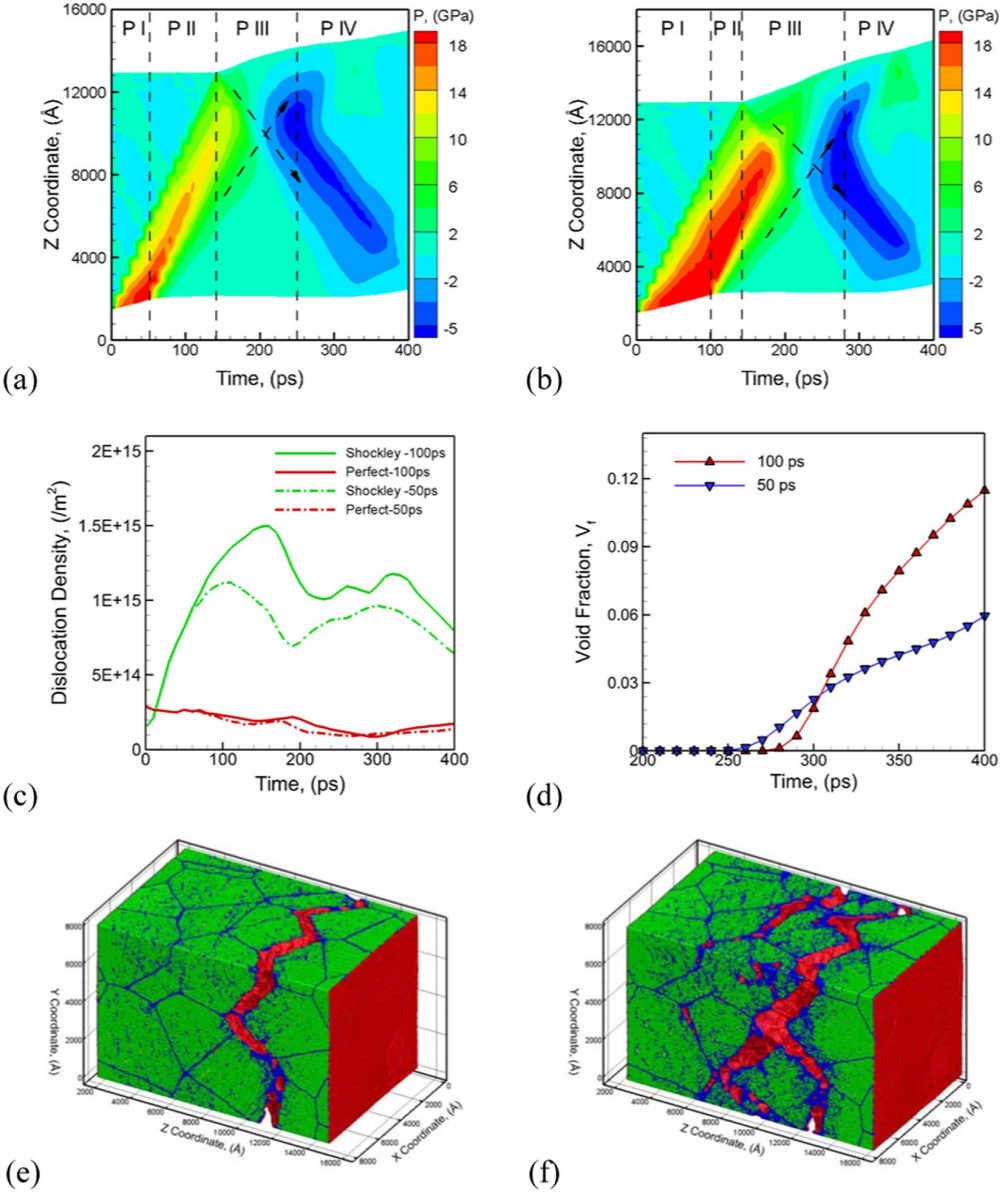
The plots for the evolution of pressure during spall failure of 400 nm grain sized polycrystalline Al system predicted by L8-QCGD simulation for a velocity of 1 km/s for the piston and pulse duration of 50 ps and 100 ps are shown in (**a**) and (**b**), respectively. The comparison of the evolution of perfect dislocations and Shockley partials for the two simulations is shown in (**c**) and the corresponding evolution of void fraction is shown in (**d**). The variations in the failure behavior is observed in the snapshots showing the microstructure for the system in (**e**) for the 50 ps pulse duration, and in (**f**) for the 100 ps pulse duration, at a time of 400 ps. The coloring of the atoms is used to identify defects, surface and stacking sequences using a combination of CNA and CSP values. The FCC stacked atoms are colored green, HCP stacked atoms are colored yellow, surface atoms are colored red and the disordered atoms are colored blue.

The second set of simulations discussed here demonstrates the role of pulse duration on spall failure behavior of a 400 nm grain sized 800 nm × 800 nm × 1.2 µm polycrystalline Al systems (atomistic system of ~45.6 Billion atoms) with L8-scaling relationships for the QCGD simulations. An inward piston velocity of 1 km/s (Z direction) is used with a pulse of 50 ps and 100 ps to investigate the role of loading pulse. Figure 4(a) and (b) compare temporal evolution of pressure as observed for pulse durations of 50 ps and 100 ps, respectively. A short pulse of 50 ps generates a short plastic wave (as indicated by the red region) that is unable to travel to the rear surface, and hence, results in lesser tensile pressures at the spall plane as compared to that for the case for a 100 ps pulse wherein the plastic wave is able to reach the rear surface. The variations in pulse duration for the piston also results in variations in the evolution of defect densities during phases ‘P-I’, ‘P-II’, ‘P-III’ and ‘P-IV’ as shown in Fig. 4(c) wherein higher densities of Shockley partials result in the metal for the 100 ps pulse. This variation in the evolution of defect densities results in variations in the strain rates generated for the simulation, the spall strength, and also the evolution of void fraction as shown in Fig. 4(d). This variation in defect density results in variations in the nucleation and growth of voids for the two systems as shown by the microstructural snapshots at a time of 400 ps. The snapshots show void nucleation and growth limited to a small width of the sample (small spall width) along the grain boundaries for the 50 ps pulse simulation in Fig. 4(e), whereas void nucleation is observed at grain interior regions in addition to at the boundaries for the 100 ps pulse (wider width of the spall plane) as shown in Fig. 4(f).

**Figure 5:**
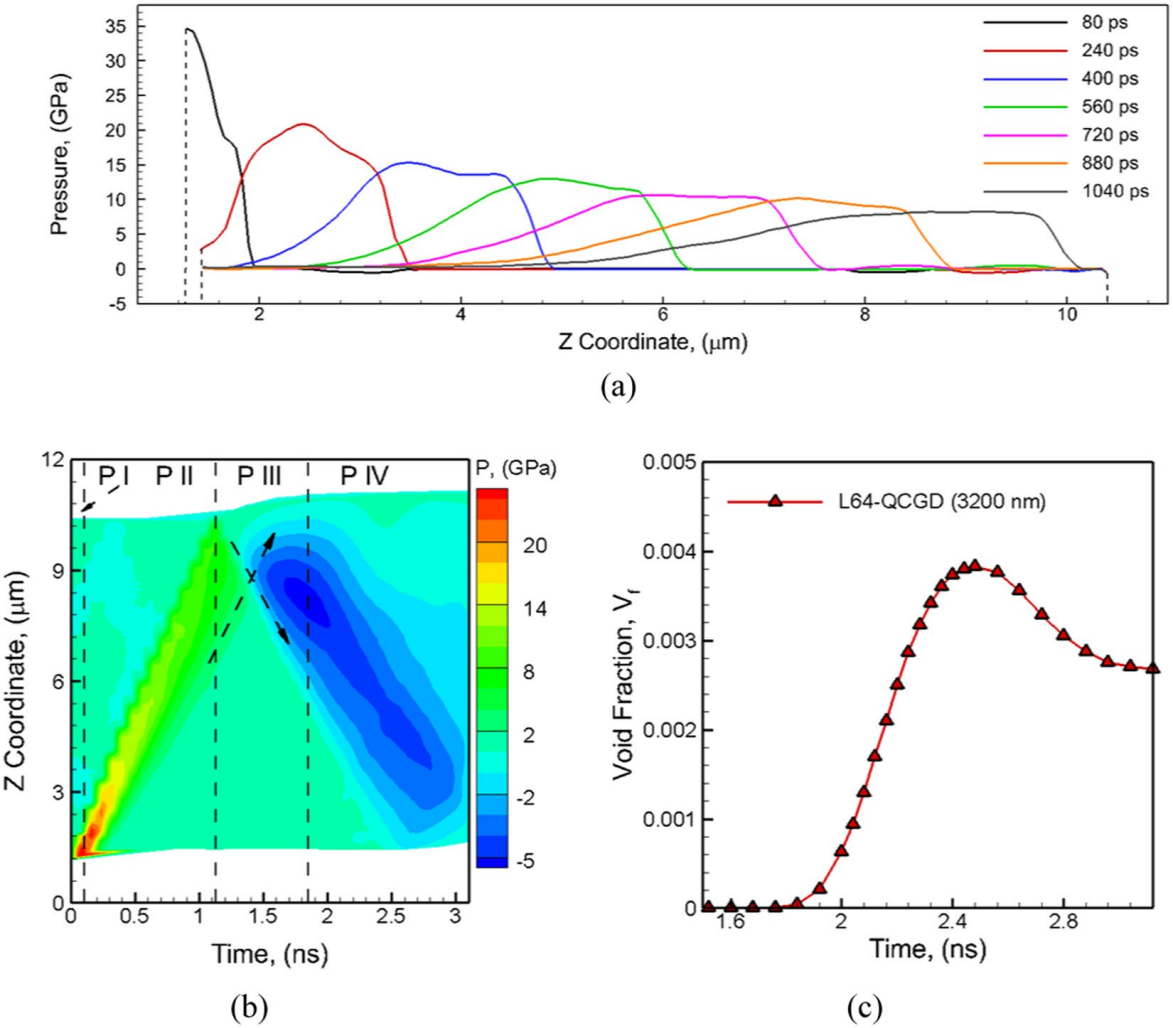
(**a**) The plots showing the decay of the compressive pressure at various times in the Z direction as the shock travels the metal for 3.2 µm grain sized polycrystalline Al system predicted by L64-QCGD simulation for a velocity of 1.5 km/s for the piston and pulse duration of 100 ps. The corresponding time evolution of (**b**) pressure and (**c**) void fraction shows the characteristics of incipient spall.

The third example demonstrates the role of the size of the sample on spall failure behavior of a 3.2 µm grain sized 6.4 µm × 6.4 µm × 9.6 µm polycrystalline Al systems (atomistic system of ~24 Trillion atoms) with L64-scaling relationships for the QCGD simulations. An inward piston velocity of 1.5 km/s (Z direction) is used with a pulse of 100 ps using a time-step of 0.1 ps to investigate the role of system size. The short duration of the shock pulse leads to a generation of a peak pressure of ~34 GPa in a very thin region of the sample. This peak pressure decays rapidly as soon as the shock wave is released into the microstructure followed by a slower relaxation of the elastic pressure as the shock wave travels through the material as shown in Fig. 5(a). The temporal evolution of pressure as observed for the 100 ps pulse is shown in Fig. 5(b). The short pulse of the system and the large system size results in a weaker state of tri-axial tensile stress that is able to nucleate voids at the grain boundaries. The nucleated voids, however, are not observed to grow continuously as the tensile wave propagates towards the piston end of the sample. As a result, the evolution of void fraction, as shown in Fig. 5(c) shows a peak value at ~2.5 ns and then decreases as the system expands under the tri-axial tensile stress without any further growth of the voids.

**Figure 6:**
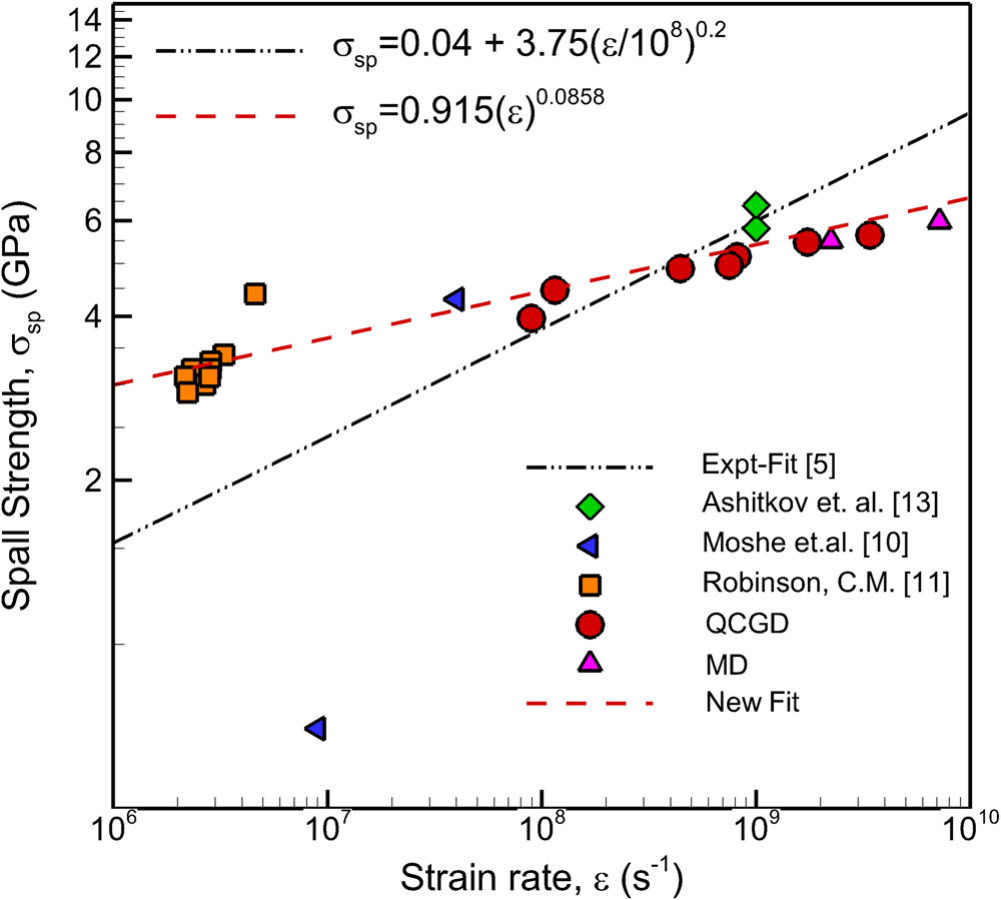
The values of spall strength computed with MD simulations (reported here), QCGD predicted “atomistic” values and experimentally calculated values^5,10,11,13^. The dashed-red line is a new strain rate dependence of the spall strength that bridges the MD predicted values with the experimental values at strain rates greater than 10^6^ s^−1^.

The QCGD simulations discussed here can be used to determine a dependence of spall strength values of various microstructures on the loading strain rates. The spall strength values from various QCGD simulations discussed in the validation (Supplemental Information S5 to S8) suggest that a self-consistent “atomic scaling factor” can be used to predict an “*atomistic spall strength”* value for any microstructure for any level of coarsening. This *“atomic scaling factor”* for the spall strength is validated by comparing the predicted atomistic spall strength for the L4-QCGD simulation of a 100 nm grain sized 0.250 µm × 0.25 µm × 0.50 µm polycrystalline Al system represented by ~29 Million R-atoms with the spall strength predicted by a MD simulation carried out using LAMMPS^42^ and comprising of ~1.85 Billion atoms for the same grain size and orientation relationships. More details about this atomic scaling factor are provided in Supplemental Information S11. The computed “*atomistic spall strength”* values for the spall strength, peak shock pressures in the spall region and the strain rates generated for the various QCGD simulations discussed above are tabulated in Table 3. The QCGD predicted variation of the spall strength of the various polycrystalline samples discussed here on loading strain rates is plotted in Fig. 6 along with the spall strength values predicted experimentally^5,10,11,13^ and using MD simulations reported here. It can be seen that the computed values of the spall strength agree with MD-predicted strain rate dependence and the experimental values. Figure 6 shows a new dependence (dashed-red line) of the spall strength on strain rates at strain rates >10^6^ s^−1^ that bridges the gap between the MD simulations and experimental data as compared to the dependence suggested experimentally (the black-dash-dot line).

**Table 3:** Calculated values of strain rate (s^−1^), spall strength (GPa), and peak compressive pressure (GPa) computed using a velocity of 1 km/s and varying pulse durations for the piston for various polycrystalline Al microstructures.

Grain Size, ***d*** (nm)	System Size (nm × nm × nm)	Shock Pulse (ps)	Peak Compressive Pressure (GPa)	Strain Rate, $\dot{\varepsilon }$ (s^−1^)	Atomistic Spall Strength, ${\sigma }^{At-Spall}$ (GPa)
50 (MD)	100 × 100 × 150	12.5	18.03	7.17 × 10^9^	5.97
100 (L2)	200 × 200 × 300	25	18.36	3.46 × 10^9^	5.64
100 (MD)	250 × 250 × 500	53	18.95	2.24 × 10^9^	5.50
200 (L4)	400 × 400 × 600	50	18.36	1.74 × 10^9^	5.47
400 (L8)	800 × 800 × 1200	100	18.62	8.17 × 10^8^	5.15
400 (L8)	800 × 800 × 1200	50	12.40	7.49 × 10^8^	4.96
800 (L16)	1600 × 1600 × 2400	200	17.96	4.43 × 10^8^	4.89
3200 (L64)	6400 × 6400 × 9600	800	20.82	1.15 × 10^8^	4.47
3200 (L64)	6400 × 6400 × 9600	100	9.51	8.93 × 10^7^	3.97

While these results demonstrate the capability of QCGD simulations to unravel the mesoscale evolution of defects/damage, it should be noted that a lower limit of coarsening exists based on the microstructure under study. The current efforts demonstrate the capability to retain the shock response characteristics for a 40 nm grain sized nanocrystalline Al system using L2-scaling relationships. This ratio of the characteristic feature size (bulk crystalline regions) is currently considered as the lower limit of coarsening for QCGD simulations for deformation behavior of a material. This translates to the lower limits for the QCGD simulations to be grain sizes (or characteristic feature sizes) of 80 nm, 160 nm, 320 nm, 0.640 µm, and 1.28 µm, for L4-, L8-, L16-, L32-, L64-scaling relationships, respectively. The lower length scales for the grain sizes, although not studied explicitly, are not recommended for coarsening as the MD simulations are able to investigate the grain size effects at these length scales. The framework of the QCGD simulations is aimed to model bulk response of a metal and hence the deformation behavior is modeled by using representative atoms and defect structures to describe collective behavior of several atoms/defects. As a result, this method is not applicable to model mesoscale behavior of individual defects (point defects or individual dislocations). Similarly, systems wherein grain boundary based processes such as nanocrystalline microstructures (with features less than 40 nm) and surface based processes (nano clusters) determine the microstructural response are likely to show deviations from the behavior predicted using MD simulations. In addition, thermally activated processes have not been investigated using QCGD simulations and will be the focus of future research directions. In addition, QCGD simulations are based on scaling relationships for interatomic potentials to determine the energetics of the R-atoms and hence also retain capabilities and limitations of interatomic potentials to model deformation and failure behavior.

Thus, the QCGD framework accelerates predictive capability of MD simulations to time and length scales that are not possible even with state-of-the-art computing resources while implicitly retaining atomic scale characteristics of the deformation and failure mechanisms observed in MD simulations. This acceleration in the capability can be estimated based on the reduction of the number of atoms (R-atoms) being modeled i.e. 1/*N*_*cg*_ and the improved time step of the simulations listed in Table S1 for various levels of coarsening. In addition, the QCGD simulations significantly reduce the amounts of data generated in the simulations that aides in the visualization and post-processing of the data and hence is a step towards reducing the “BIG DATA” being generated using MD simulations. The current capabilities of the QCGD simulations have been extended to the HCP systems (Ti) to model the kinetics of melting (melt front propagation), the pressure-temperature phase diagram, and the shock response and spall failure of Ti microstructures^43^.

## Conclusions

The QCGD simulations discussed here demonstrate the capability to model shock response and spall failure behavior of polycrystalline Al systems for grain sizes ranging from 50 nm to 3.2 µm using various levels of coarsening. The QCGD microstructures for higher levels of coarsening correspond to system sizes that are well beyond current capabilities of MD simulations using state-of-the-art computing resources. The QCGD simulations use representative atoms in a coarse-grained (CG) microstructure to model the collective dynamics of several atoms using scaled interatomic potentials. This framework incorporates the plastic deformation and failure mechanisms using representative defect structures (dislocations, vacancies, faults, etc.) to describe the collective nucleation and evolution of dislocation densities in the CG microstructure. The framework based on scaling relationships is implicitly able to retain atomic scale mechanisms related to the nucleation, dissociation, recombination of dislocations as well as reactions for the formation of Stair-rods, Hirth-locks, etc. as observed in MD simulations during shock propagation and spall failure of polycrystalline microstructures. The QCGD simulations also retain the MD-predicted shock wave velocities as well as the evolution of temperature in the metal due to plastic deformation behind shock fronts and during damage evolution during spall failure. The QCGD simulations are able to quantify the rate of evolution of void fractions as a function of loading strain rates as well as identify the role of evolution of dislocation densities on the deformation and spall failure behavior. The simulations carried out to investigate the effect of microstructure, shock pulse and system dimensions provide critical insights in the microstructural evolution that needs to be incorporated in continuum models to be able to predict the shock deformation and spall failure behavior of polycrystalline metals. One of the consequences of the collective energetics for nucleation and evolution of dislocations is slight strengthening of CG systems. However, a self-consistent scaling factor is observed for shock loading simulations. An atomic scaling factor is defined that can determine a corresponding “*atomistic spall strength*” value for any level of coarsening and any microstructure based on the values predicted using QCGD simulations. The QCGD simulations are able to define a new strain rate dependence of the spall strength that bridges the gap between MD-predicted values and the experimental values at strain rates greater than 10^6^ s^−1^. The QCGD simulations can thus unravel the evolution of defects/damage in polycrystalline microstructures at the mesoscales and complement the experimental efforts towards the design of damage tolerant materials.

## Methods

### The Quasi-Coarse-Grained Dynamics (QCGD) Method

The QCGD method uses representative atoms (R-atoms) to coarse-grain an atomistic microstructure and the equations of motion are solved for the R-atoms that model the collective energetics of several atoms. Such a coarse-grained structure is created by the representation of a volume of *n*×*n*×*n* atomic scale unit cells using 1 “coarse-grained unit cell” (CG-cell) with the lattice constant defined based on the level of coarsening. The scaling relationships comprise of a “*distance scaling parameter”*, ${A}_{cg}=n$ and a “*number of atoms coarse-grained parameter”*, ${N}_{cg}=n\times n\times n$ to retain the MD-predicted energetics of a $n\times n\times n$ system using 1 CG-cell. The atomic scale interatomic potential is scaled using ${A}_{cg}$ to retain the atomic scale energies for R-atoms in CG microstructures. The energies and degrees of freedom of the R-atom are then scaled by ${N}_{cg}$ to incorporate the collective atomic scale dynamics of ${N}_{cg}={n}^{3}$ atoms. These parameters (as discussed in Supplemental Information: S1) allow the modeling of the microstructure using significantly less number of R-atoms and also allow significantly larger values for the allowed time-steps. The scaling behavior retains the atomic scale characteristics of the MD predicted deformation and phase transformation phenomena^37^.

### Polycrystalline Microstructures

The initial microstructures are generated using the “*Voronoi construction method*”^44^ with periodic lateral directions (X, Y) and the loading direction (Z) is kept free.

### Interatomic Potentials

The QCGD simulations are carried out with various levels of coarsening using scaled interactions between R-atoms based on the “embedded atom method” (EAM) potential for Al^45^.

### Defect (Dislocation) Characterization

The “dislocation extraction algorithm” (DXA)^46,47^ is used to characterize the various types of dislocations in the metal. The snapshots generated during the MD and QCGD simulations are analyzed using the “centrosymmetry parameter” (CSP)^48^ and “common neighbor analysis” (CNA)^49^ to identify various defect structures and surfaces (voids) during the simulations.

### Shock Setup and Pressure Analysis

The validation of the shock response is based on impact simulations in the polycrystalline systems using a rigid piston at one end of the sample (bottom) that is driven inward for a given pulse duration (square pulse) with a constant inward velocity (Z direction), *U*_*p*_ of 1 km/s (piston velocity). The piston impact generates a planar shock wave in the metal that results in the nucleation and evolution of defects. The shock wave reflects back as a tensile wave from the rear surface and interacts with the tail that is moving towards the rear surface. A tri-axial stress state is generated and nucleates multiple voids initiating the spall failure of the metal. The temporal evolution of the pressure in the system is used to analyze the wave velocities, interactions, and also evaluate the spall strength of the metal for the loading conditions generated^31,32,34,35^.

## Electronic supplementary material

Supplemental Information
