# Supplementary material for: The Quasi-Coarse-Grained Dynamics Method to Unravel the Mesoscale Evolution of Defects/Damage during Shock Loading and Spall Failure of Polycrystalline Al Microstructures
Source: Sci Rep. 2017 Sep 28;7:12376. doi: 10.1038/s41598-017-12340-4 (PMC5620078; doi:10.1038/s41598-017-12340-4)
Supplement: Supplementary file 1 — Supplemental Information [file 41598_2017_12340_MOESM1_ESM.pdf]

SUPPLEMENTAL INFORMATION, FIGURES AND FIGURE CAPTIONS

Title: The Quasi-Coarse-Grained Dynamics Method to Unravel the Mesoscale Evolution of Defects/Damage during Shock Loading and Spall Failure of Polycrystalline Al Microstructures

Garvit Agarwal[1], *Ramakrishna R. Valisetty*[2], *Raju R. Namburu*[2], *Arunachalam M. Rajendran*[3], *and Avinash M. Dongare*[1,*]

[1] Department of Materials Science and Engineering and Institute of Materials Science, University of Connecticut, Storrs, Connecticut 06269, USA

[2] Computational and Information Sciences Directorate, U.S. Army Research Laboratory, Aberdeen Proving Ground, Maryland 21005, USA

[3] Department of Mechanical Engineering, University of Mississippi, 201-B Carrier Hall, University, MS 38677, USA

*** Corresponding Author:**

Avinash M. Dongare

Materials Science and Engineering, University of Connecticut, Storrs, CT-06269

Email: dongare@uconn.edu

S1: The Quasi-Coarse-Grained Dynamics (QCGD) Method

The QCGD method [37] is based on solving the dynamics of reduced number of representative atoms (R-atoms) and improved time steps to extend the capability of molecular dynamics (MD) simulations to the mesoscales. Such a reduction of the number of atoms is achieved by using coarse-grained unit cells (CG-cell) with the lattice constant defined based on the level of coarsening. For example, a system of $n \times n \times n$ atomic scale unit cells is modeled using 1 coarse-grained unit cell and reduces the number of R-atoms by $N_{cg} = n^3$ in the QCGD framework as level "L*n*" of coarsening. Such a representation retains the symmetry and neighborhood structure of the R-atoms as would be in the atomic scale microstructure. The atomic scale interatomic potential is then scaled by a "distance scaling parameter", $A_{cg} = n$ that retains the energy of the R-atom as would be in the atomic scale microstructure. The energies and the degrees of freedom of the R-atom are then scaled by $N_{cg} = n^3$ to incorporate the collective dynamics (energies and degrees of freedom) of the missing atoms in the CG-microstructure. The QCGD parameters and the time step of the simulations for the various levels of coarsening are listed in Table S1.

Table S1: Scaling parameters and time-steps used for each level of coarsening used in QCGD simulations. The allowable time-steps are chosen here as the highest values of the time-step that not-only render conservation of energy in the QCGD simulations for the NVE ensemble, but also retain the MD-predicted shock profiles for the same microstructure.

QCGD Level of Coarsening	Atomic Unit Cells epresented $n \times n \times n$	Distance Scaling Parameter (A_{cg})	Number of Atoms Represented (N_{cg})	Time-step
L1 (MD)	$1 \times 1 \times 1$	1	1	02 fs
L2-QCGD	$2 \times 2 \times 2$	2	8	10 fs
L4-QCGD	$4 \times 4 \times 4$	4	64	24 fs
L8-QCGD	$8 \times 8 \times 8$	8	512	48 fs
L16-QCGD	$16 \times 16 \times 16$	16	4096	80 fs
L64-QCGD	$64 \times 64 \times 64$	64	262,144	100 fs

These scaling parameters (A_{cg} and N_{cg}) thus incorporate the collective dynamics of an atomic scale system of $n \times n \times n$ unit cells by using reduced number of R-atoms and improved time steps and are able to reproduce the structural energetics, the deformation behavior, as well as the phase transformation behavior predicted using MD simulations. More details about the functional formulation and scaling factors can be found in Reference [37]

S2: Consequences of the QCGD Scaling Relationships

While the scaling relationships retain the atomic scale energetics of the R-atoms, the representation of the collective dynamics of $N_{cg} = n^3$ atoms in the QCGDframework renders consequences on the computed total energetics of the system that affects only the values of the energies (cohesive energy, surface energy, grain boundary energy, etc.) predicted by the QCGD

simulations and does not affect the dynamics of the R-atoms. These consequences are discussed below**.**

The first consequence of the scaling relationships is the scaling of cohesive energies of Al with the level of coarsening. A comparison of the computed equation of state (EOS) for Al as predicted using MD, L2-QCGD and L4-QCGD simulations for the same volume of the system is shown in Figure S1(a), (b), and (c), respectively. The L2-QCGD predicted values for cohesive energy (total energy divided by total number of atoms/R-atoms) are 8 times the MD predicted value as shown in Figure S1(b) and the L4-predicted values are 64 times the MD predicted values as shown in Figure S1(c). This scaling is attributed to the scaling factor of $N_{cg} = 8$ in the L2-QCGD simulations and $N_{cg} = 64$ in the L4-QCGD simulations used to scale the energy of a R-atom to incorporate the collective dynamics of missing atoms in the CG systems.

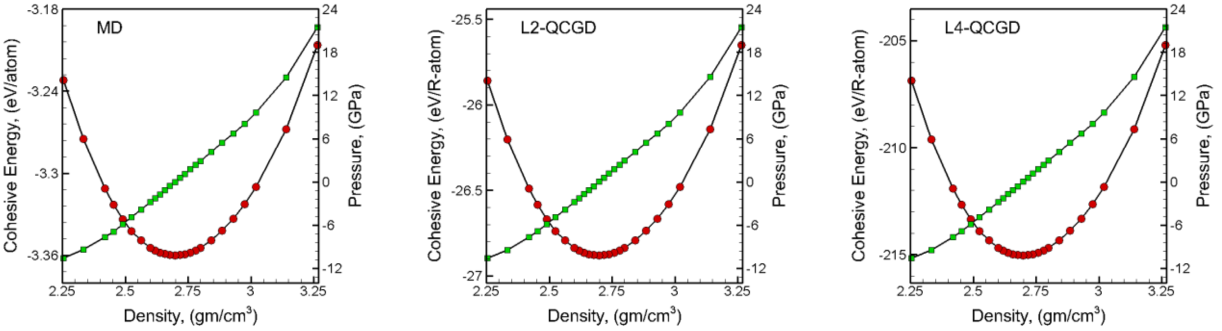

(a) (b) (c)

Figure S1: Plots of cohesive energy (red circles) and corresponding pressure (green diamonds) are shown as a function of density predicted for the MD simulation in (a), for the L2-QCGD simulation in (b), and for the L4-QCGD simulations in (c) for EAM Al.

Table S2: Surface energies (mJ/m^2) for the (100), (110) and (111) surfaces as calculated using MD, L2-QCGD and L4-QCGD.

Surface	MD (mJ/m^2)	L2-QCGD (mJ/m^2)	L4-QCGD (mJ/m^2)
(100)	942.508	1884.804	3769.608
(110)	1004.743	2009.486	4018.829
(111)	869.765	1739.178	3478.005

The second consequence of the scaling relationships is the scaling of the energetics of the surfaces and grain boundaries. To investigate the scaling factors, the surface energies for the (100), (110) and (111) surfaces are calculated using MD, L2-QCGD and L4-QCGD for the same total volume of the system. The MD system used comprises of $40\times40\times40$ unit cells and is represented using L2-QCGD and L4-QCGD systems comprising of $20\times20\times20$ CG-cells and $10\times10\times10$ CG-cells, respectively. The snapshots of these surfaces after relaxation showing the atoms/R-atoms colored based on the values of the total energy are shown in Figure S2. The R-atoms retain the MD-predicted energies at the surface, beneath the surface, and in the bulk. This retaining of the atomic scale energy, however, will result in a scaling of the surface energy in the QCGD simulations due to the scaling parameter N_{cg}. The calculated values showing the scaling of the surface energy values for the three different surface planes are listed in Table S2. Thus, the

QCGD predicted surface energy scales with the level of coarsening. A similar scaling is calculated for energies of grain boundaries wherein the QCGD simulations retain the MD-predicted total energy of an R-atom at the grain boundary and in the bulk. However, as will be discussed later, such a scaling behavior of energies does not affect the deformation behavior and spall failure behavior of polycrystalline metals under shock loading conditions.

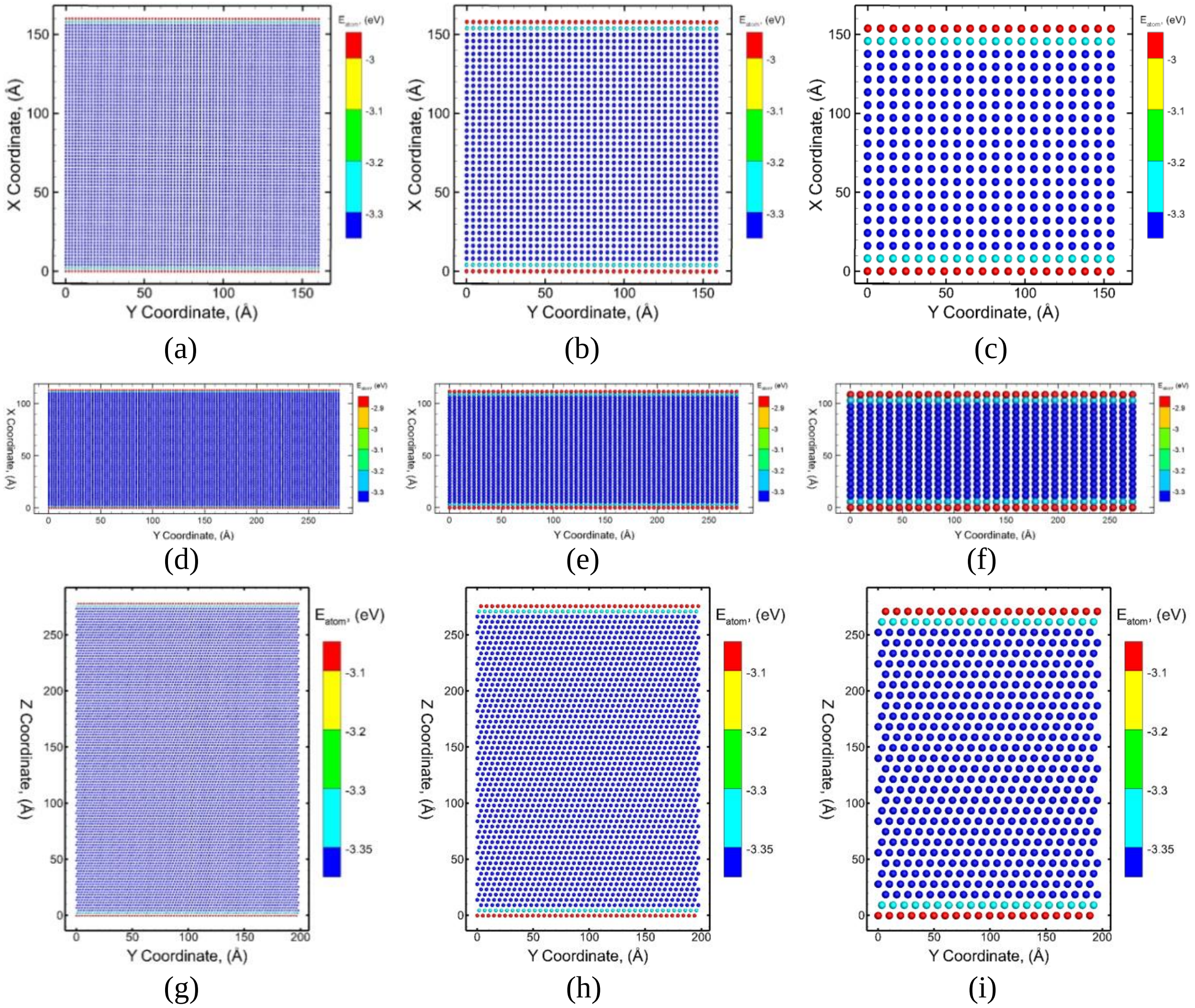

Figure S2: The atomic scale microstructure of Al systems with the (100), (110) and (111) surface is shown in (a), (d), and (g), respectively. The corresponding representative microstructures using L2-scaling for the (100), (110) and (111) surface are shown in (b), (e) and (h), respectively. Similarly, the corresponding representative microstructures using L4 scaling for the (100), (110) and (111) surface are shown in (c), (f) and (i), respectively. The color of the atoms corresponds to values for total energy.

S3: Collective Dynamics of Defects in QCGD Simulations

An important aspect in the capability of QCGD simulations to model shock response is the capability to reproduce the atomic scale characteristics of nucleation and evolution of dislocations. This capability is incorporated through modeling the collective evolution of defects

rather than modeling individual defects using representative defect structures. This collective evolution is reflected in the formation energies of the defects. For example, a missing R-atom in the CG microstructure corresponds to $N_{cg} = n^3$ missing atoms in MD simulations. Thus, one missing R-atom in the L4-QCGD simulation corresponds to $N_{cg} = 64$ missing atoms in the MD simulations. This scaling renders a vacancy formation energy in the QCGD simulations that is scaled by $N_{cg} = n^3$ times the MD predicted value and corresponds to a collective representation of vacancies in MD simulations.

Table S3: Calculated values for stacking fault width and stacking fault energy for L2-QCGD and L4-QCGD simulations by the addition of two half planes of R-atoms in the system and the corresponding atomistic values predicted using MD simulations for the same system volume.

QCGD level	QCGD Predicted Values		MD Predicted Values		
	Stacking Fault Width (Å),	Stacking Fault Energy (mJ/m^2)	Number of stacking Faults	Stacking Fault Width (Å),	Stacking Fault Energy (mJ/m^2)
L2-QCGD	24.308	214.499	2	12.130	107.461
L4-QCGD	48.802	427.361	4	12.130	107.461

A similar scaling behavior is observed for the stacking faults in the QCGD simulations. The nucleation and evolution of dislocations/faults in QCGD simulations represents a collective evolution of dislocations in an atomistic simulation. To demonstrate this collective description of the stacking faults/dislocations, a stacking fault is created in a L2-QCGD system by the addition of two (110) half planes and relaxed to have a stacking fault on the (111) glide plane that separates two Shockley partial dislocations. The computed width of the stacking fault and the stacking fault energy (based on the stacking fault width) for the L2-QCGD systems is calculated to be 214.499 mJ/m^2. The value predicted for the stacking fault energy in MD simulation is 107.461 mJ/m^2 and results in a stacking fault width of 12.13 Å. Thus, the stacking fault width and stacking fault energy predicted by L2-QCGD simulation is calculated to be 2 times the values predicted using MD simulations. However, the two additional (110) half planes in L2-QCGD simulation correspond to the addition of 4 additional (110) half planes of atoms in the atomic scale system (to have an exact scaling of the atoms i.e. 8 times that of L2-QCGD system). A comparative MD simulation is therefore carried out for a system with 4 additional (110) half planes and the energy minimized structure shows formation of two stacking faults each with half of the width predicted by the L2-QCGD simulation. The snapshots showing one large L2-QCGD stacking fault and the two MD stacking faults are shown in Figure S3(a) and (b), respectively. The combined width of the two faults and the stacking fault energy as listed in Table S3 compares very well with the width and energy of the one stacking fault in the L2-QCGD simulation. Thus, the nucleation of a stacking fault in FCC Al in the L2-QCGD simulations corresponds to the collective nucleation of two stacking faults in the MD simulations. A similar behavior is observed for the L4-QCGD simulations, wherein nucleation of one stacking fault is observed with a width that is four times the MD predicted value and corresponds to a collective nucleation of four stacking faults in the MD simulations. Comparison snapshots showing the one large L4-QCGD stacking fault and corresponding four MD stacking faults are shown in Figure S3(c) and (d), respectively. A similar scaling relationship exists for the stacking fault energy for the higher levels of coarsening in the QCGD simulation.

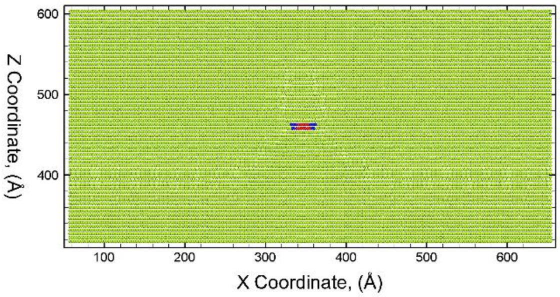

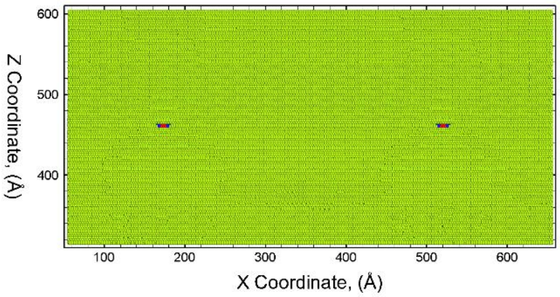

(a) (b)

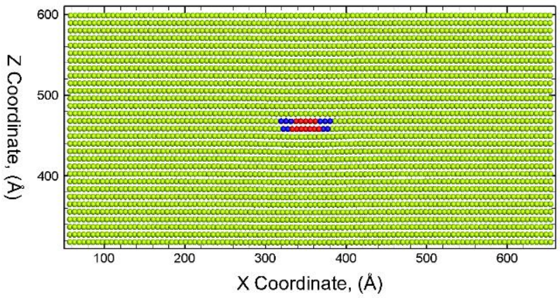

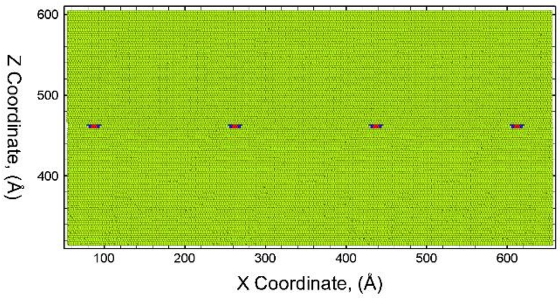

(c) (d)

Figure S3: (a) The microstructure in the L2-QCGD simulation showing a stacking fault created by the introduction of two additional half planes of R-atoms. (b) The corresponding MD simulation showing the stacking faults created due to the introduction of four additional half planes of atoms. Similarly, the microstructure in the L4-QCGD simulation showing a stacking fault created for the L4-QCGD simulation due to the introduction of two additional half planes of R-atoms is shown in (c) and the corresponding MD simulation showing the stacking faults created due to the introduction of eight additional half planes of atoms is shown in (d) Green color represent FCC stacking, red color represent HCP stacking (stacking fault) and blue color represent a disordered structure (dislocation core in these snapshots) for the atoms.

Thus, the nucleation of one large stacking fault in L2-QCGD simulations represents the collective nucleation of two stacking faults in the MD simulations and hence requires larger energies to nucleate defects. Similarly, the nucleation of one large stacking fault in L4-QCGD simulations represents the collective nucleation of four stacking faults in the MD simulations and hence requires larger energies to nucleate defects as compared to L2-QCGD simulations. A similar scaling behavior of defect energetics is observed for higher levels of coarsening. As a result, the scaling relationships and the collective description results in a slight strengthening of the system as compared to the atomistic system and the amount of strengthening scales with the level of coarsening. Thus, the defects in the QCGD simulations, although lower in number represent accurate collective atomic scale energetics of several defects in the MD simulations. Such a representation is reasonable when the systems/phenomena modeled will result in defects that are significantly large in number that a collective description is a good approximation. This is demonstrated by the capability of the QCGD simulations to retain the microstructural evolution under shock loading conditions i.e. the various dislocation reactions such as dissociation of perfect dislocations to Shockley partials, formation of stair-rods, Hirth-locks, etc. observed under shock loading in MD simulations.

S4: Scaling Relationships to Predict Atomic Level Dislocation Densities

The pre-existing dislocation density for the various types of dislocations in the initial systems created for the various levels of coarsening are tabulated in Table S4. It can be seen that while the coarsening of the microstructure does not retain the exact dislocation densities, the various levels of coarsening retain the relative fractions of the various types of pre-existing dislocations at the grain boundaries for the polycrystalline systems.

Table S4: Initial dislocation densities for various types of dislocations in the initial polycrystalline Al microstructures modeled using MD and the various levels of the QCGD simulations for the same grain centers and grain orientation relationships. The 50 nm system is created using atoms (MD) and L2-scaling parameters for R-atoms in the QCGD simulations. Similarly, the 100 nm grain size system is created using L2-scaling and L4-scaling parameters for R atoms, the 200 nm grain size system is created using L4-scaling and L8-scaling parameters for R-atoms and the 400 nm grain size system is created using L8-scaling and L16-scaling parameters.

Grain Size	Level of Coarsening	Perfect x 10^{14} (m^{-2})	Shockley x 10^{14} (m^{-2})	Stair-rod x 10^{14} (m^{-2})	Frank x 10^{14} (m^{-2})	Hirth x 10^{14} (m^{-2})	Total x 10^{14} (m^{-2})
50 nm	MD	220.9	129.3	0.10	14.7	0.045	365.0
	L2-QCGD	77.7	45.8	0.009	6.02	0.016	129.5
100 nm	L2-QCGD	29.11	14.95	0.017	1.97	0.0034	46.06
	L4-QCGD	11.75	7.69	0.010	0.97	0.0057	20.43
200 nm	L4-QCGD	7.157	4.062	0.0020	0.505	0.00054	11.728
	L8-QCGD	3.067	2.11	0.00076	0.248	0.0013	5.431
400 nm	L8-QCGD	2.895	1.561	0.0014	0.198	0.00022	4.657
	L16-QCGD	1.153	0.763	0.00016	0.098	0.00057	2.016

A comparison of the values of the dislocation density suggests that an '*atomic scaling factor*' can be defined to predict a '*representative atomistic dislocation density*' based on the values computed for the various levels of coarsening. For example, the atomic scaling factor for the dislocation density predicted in L2-QCGD structures is defined as the ratio of the densities predicted by MD and L2-QCGD simulations as $SCF^{\rho}_{MD/L2} = \rho_{MD} / \rho_{L2}$ for the same microstructure and system size using the same grain centers and orientation relationships. Similarly, a '*representative atomic level dislocation density*' for the higher levels of coarsening can be computed based on the comparative fractions of the dislocation densities. These fractions are obtained using comparisons for the L4-QCGD, L8-QCGD and L16-QCGD systems using the density for the L2-QCGD, L4-QCGD and L8-QCGD systems, respectively. These fractions are written as

$$\rho_{MD/L2} = \rho_{MD} / \rho_{L2} \quad (1a)$$

$$\rho_{L2/L4} = \rho_{L2} / \rho_{L4} \tag{1b}$$

$$\rho_{L4/L8} = \rho_{L4} / \rho_{L8} \tag{1c}$$

$$\rho_{L8/L16} = \rho_{L8} / \rho_{L16} \tag{1d}$$

These fractions for dislocation density values are calculated based on ratios of dislocation densities tabulated in Table S4 and are used to compute a '*representative atomic level dislocation density*' for the various levels of coarsening as

$$\rho_{L2}^{Atomic} = \rho_{L2} \cdot \rho_{MD/L2} \tag{2a}$$

$$\rho_{L4}^{Atomic} = \rho_{L4} \cdot \rho_{MD/L2} \cdot \rho_{L2/L4} \tag{2b}$$

$$\rho_{L8}^{Atomic} = \rho_{L8} \cdot \rho_{MD/L2} \cdot \rho_{L2/L4} \cdot \rho_{L4/L8} \tag{2c}$$

$$\rho_{L16}^{Atomic} = \rho_{L16} \cdot \rho_{MD/L2} \cdot \rho_{L2/L4} \cdot \rho_{L4/L8} \cdot \rho_{L8/L16} \tag{2d}$$

For each of the grain sizes chosen for validation (50 nm, 100 nm, 200 m, and 400 nm), the predicted atomic level dislocation densities for the various types of pre-existing dislocations using the scaling factors above are tabulated in Table 1.

S5. Validation of Scaling Relationships for L2-QCGD Shock Simulations

The validation is first carried out for the L2-QCGD simulation based on the prediction of the shock wave velocities, shock pressures, defect evolution, temperature evolution and spall strength for a 50 nm grain sized polycrystalline Al system using MD simulations. The polycrystalline system comprises of dimensions of 100 nm x 100 nm x 150 nm and the same grain orientation relationships as in the MD simulations. This system corresponds to a size of ~89 Million atoms for the MD simulation and is represented by ~11 Million R-atoms using L2-scaling. A time-step of 0.002 ps is used for both the simulations. A rigid piston at one end of the sample (bottom) is driven inward for a pulse duration of 12.5 ps (square pulse) with an constant inward velocity (Z direction), U_p of 1 km/s (piston velocity). The shock velocities and spall strength is investigated by computing the average stresses along the length of the sample at various times during the simulation. A comparison of the MD and L2-QCGD predicted temporal evolution of pressure in the system along the length of the sample in the shock direction is shown in Figure S4 (a) and (b), respectively. The pressure in the system is computed as $\mathrm{P} = -\frac{1}{3}(\sigma_{XX} + \sigma_{YY} + \sigma_{ZZ})$, where σ_{XX}, σ_{YY}, and σ_{ZZ} are the average values of the stresses in X, Y and Z directions, respectively. These plots enable the investigation of the shock wave propagation (red indicates compressive pressure), reflection and interactions to generate a triaxial tensile wave (blue indicates tensile pressure). The discussion of the wave propagation behavior can be focused on the four phases of wave propagation behavior: Phase I (PI) corresponds to propagation of compression wave for the given pulse duration; Phase II (PII) corresponds to the arrival of the tail of the compressive wave and propagation of the compression wave till it reaches the rear surface; Phase III (PIII) corresponds to the expansion of the rear surface to generate a reflected tensile wave and interaction of the reflected tensile wave with the tail (as shown by the arrows) to create peak tensile pressures; and Phase IV (PIV) corresponds to the

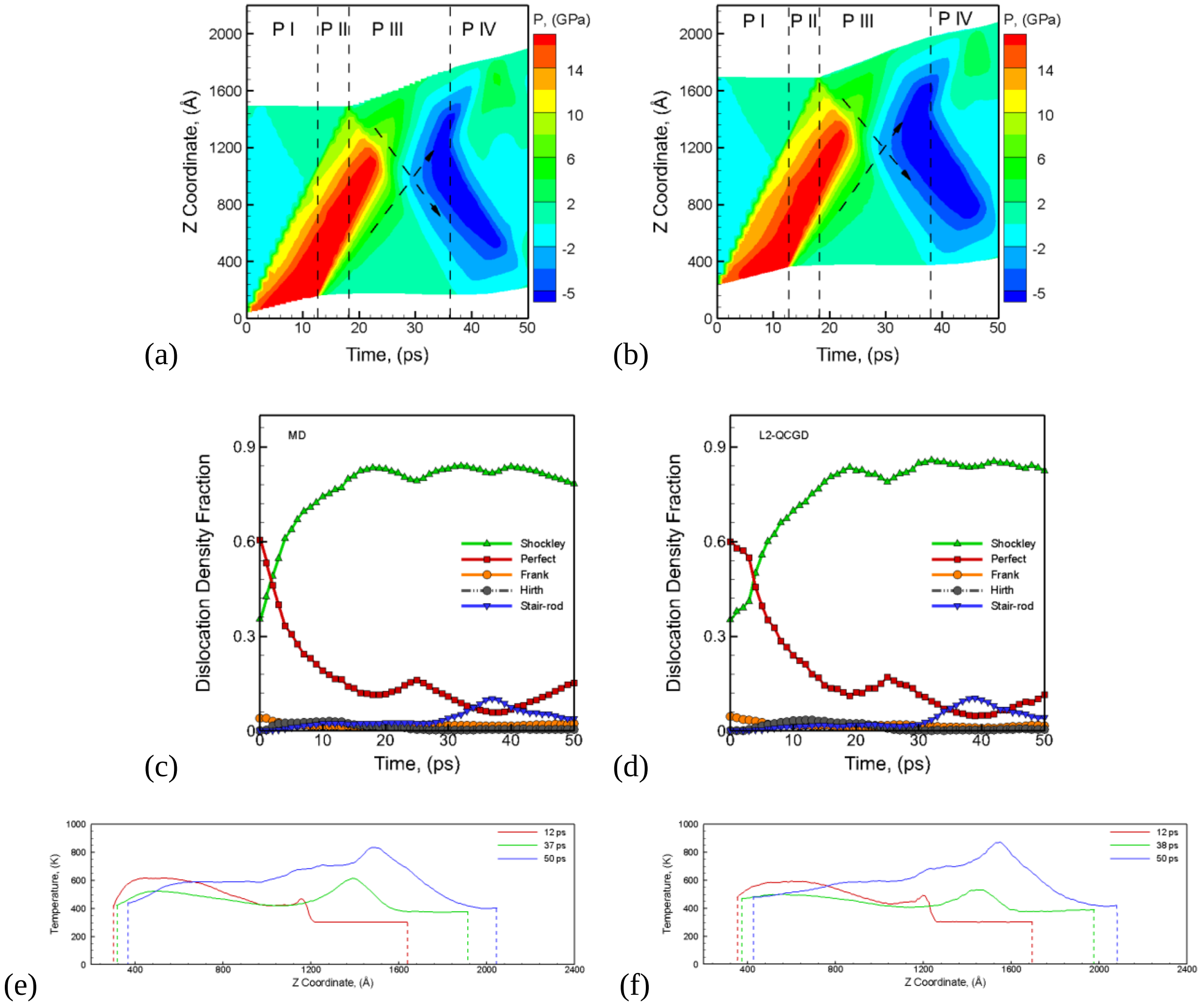

Figure S4: The plots for the evolution of pressure as a function of time during spall failure of 50 nm grain sized polycrystalline Al for a velocity of 1 km/s for the piston as predicted by the (a) MD and (b) L2-QCGD simulation. A comparison of the fractions for the different types of dislocations as a function of time is shown in (c) and (d), and corresponding evolution of the temperature at various times for the two simulations is shown in (e) and (f).

nucleation of voids that grow as the triaxial tensile wave travels towards the piston. The plots show that the L2-QCGD predicted shock wave propagation (red indicates compressive pressure), reflection and interactions to generate a triaxial tensile wave (blue indicates tensile pressure) match very well with the MD predicted wave propagation behavior. A closer look at the data suggests that the L2-QCGD spall strength (6.266 GPa) is slightly higher than that obtained using MD simulations (5.959 GPa). This slightly over-predicted value of the spall strength for the L2-QCGD simulations is attributed to the scaling of the defect energies discussed in S3 above.

In addition, a comparison of the evolution of the fraction of the various types of dislocations during the MD simulation and the L2-QCGD simulation is shown in Figure S4(c)

and (d), respectively. The MD simulation predicts a decrease in fractions of the perfect dislocations and an increase in the fraction of the Shockley partials during Phase I (PI) and includes dissociation of perfect dislocations to the Shockley partials. Phase II results in a decrease in the rate of evolution of the dislocation density fractions as the tail appears and propagates towards the rear surface. The expansion of the rear surface during Phase III results in a decrease in the fractions of the Shockley partial dislocations and a simultaneous increase in the perfect dislocation fractions attributed to the recombination of the Shockley partials to create perfect dislocations. This recombination to create perfect dislocations continues during Phase III till the reflected tensile wave interacts with the tail to create triaxial tensile stresses in the metal (as shown by arrows). The triaxial tensile stresses result in the dissociation of perfect dislocations to nucleate Shockley partials, which further interact to form stair rods during Phase III. The formation of stair-rods continues to reach a peak value at the end of Phase III i.e at the onset of spall failure. The nucleation of voids results in a decrease in the fraction of stair-rods and an increase in perfect dislocations formed by the recombination of the Shockley partials.

The L2-QCGD simulations reproduce the MD predicted characteristics related to the nucleation and evolution of dislocation density fractions. In particular, the dissociation of perfect dislocations during PI, recombination of Shockley partials during PII, formation of stair-rods during PIII, and the recombination of Shockley partials during PIV is accurately captured by the L2-QCGD simulation. Thus, the QCGD simulations retain the MD predicted atomic scale mechanisms of the dislocation nucleation and reactions that determines the shock response and spall failure behavior of the polycrystalline metal. Similarly, a comparison of the MD and L2-QCGD predicted evolution of temperature in the system due to plastic deformation at the end of shock pulse (12.5 ps), at peak tensile pressure (26 ps) and after initiation of failure (30 ps) is shown in Figure S4 (e) and (f), respectively. These results validate the capability of the L2-QCGD simulations to model the shock response of the polycrystalline system for a grain size of 50 nm and larger and retain the atomistic nature of the nucleation, evolution and interaction of dislocations.

S6. Validation of Scaling Relationships for L4-QCGD Shock Simulations

Similarly, the L4-QCGD simulations are validated by reproducing the shock response of a 100 nm grain sized polycrystalline Al system using L2-QCGD simulations. This system comprises of dimensions of 200 nm x 200 nm x 300 nm (atomistic system of ~712 Million atoms) and is represented by ~89 Million R-atoms for the L2-QCGD simulation and ~11 Million R-atoms using L4-scaling in the QCGD simulations. A time-step of 0.010 ps is used for both the simulations. A comparison of the predicted temporal evolution of pressure in the system for the L2-QCGD and L4-QCGD simulation is shown in Figure S5 (a) and (b), respectively for a piston velocity of 1 km/s and a pulse of 25 ps. The predicted spall strength value for the L4-QCGD simulation (6.20 GPa) compares very well (slightly over-predicted as observed for the L2-QCGD vs MD) with the L2-QCGD predicted value (5.934 GPa). In addition, a comparison of the evolution of the fraction of the various types of dislocations during the L2-QCGD simulation and the L4-QCGD simulation is shown in Figure S5(c) and (d), respectively. The L4-QCGD simulations reproduce the L2-QCGD predicted characteristics related to the nucleation and evolution of dislocation fractions. In particular, the dissociation of perfect dislocations during PI, recombination of Shockley partials during PII, formation of stair-rods during PIII, and the recombination of Shockley partials during PIV is accurately reproduced by the L4-QCGD

simulation. Similarly, a comparison of the predicted evolution of temperature in the system due to plastic deformation at the end of shock pulse (25 ps), at peak tensile pressure (80 ps) and after initiation of failure (100 ps) for the two simulations is shown in Figure S5 (e) and (f). These results validate the capability of the L4-QCGD simulations to model the shock response of the polycrystalline system for a grain size of 100 nm and larger and retain the atomistic nature of the nucleation, evolution and interaction of dislocations.

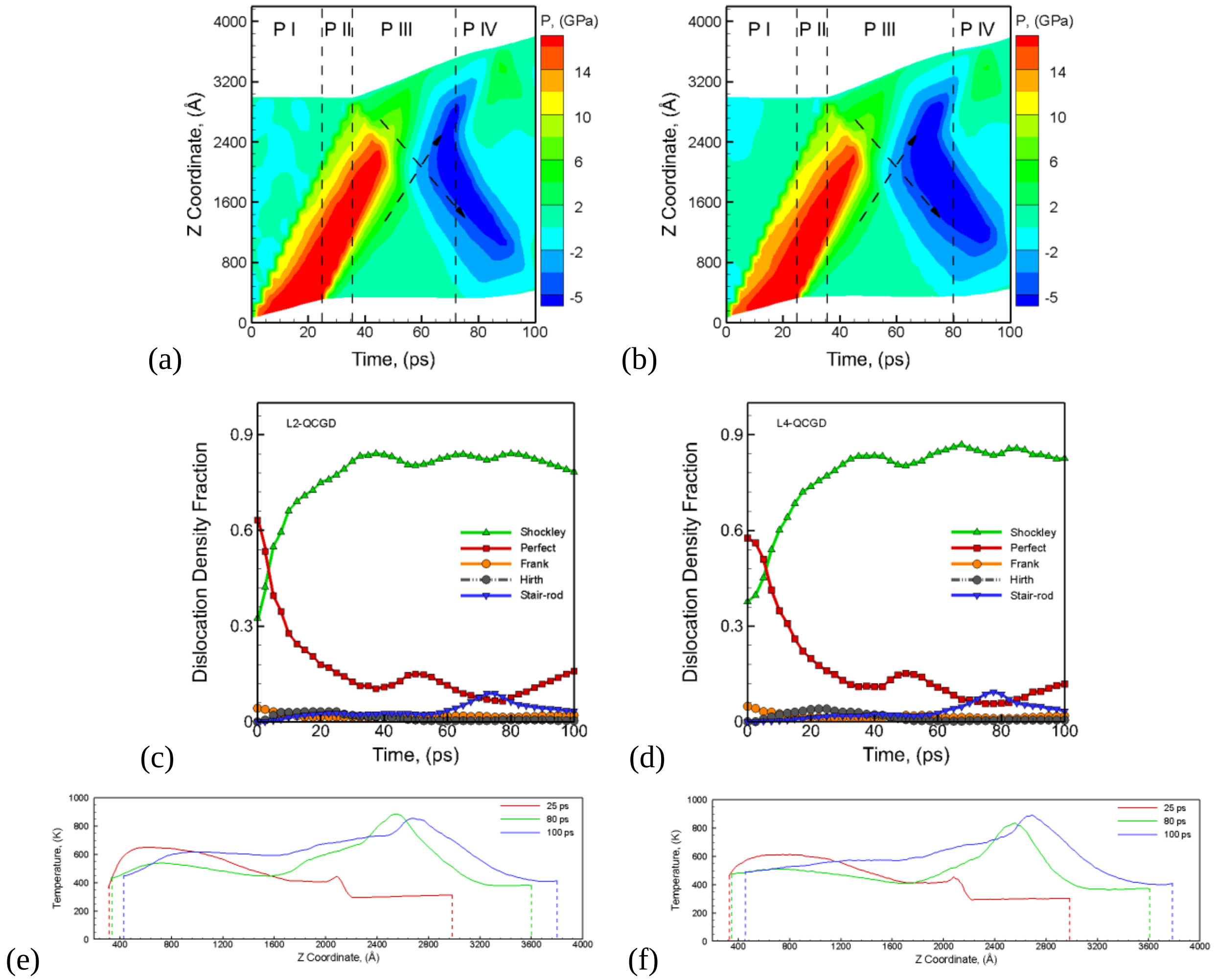

Figure S5: The plots for the evolution of pressure as a function of time during spall failure of 100 nm grain sized polycrystalline Al for a velocity of 1 km/s for the piston as predicted by the (a) L2-QCGD and (b) L4-QCGD simulation. A comparison of the fractions for the different types of dislocations as a function of time is shown in (c) and (d), and corresponding evolution of the temperature at various times for the two simulations is shown in (e) and (f).

S7. Validation of Scaling Relationships for L8-QCGD Shock Simulations

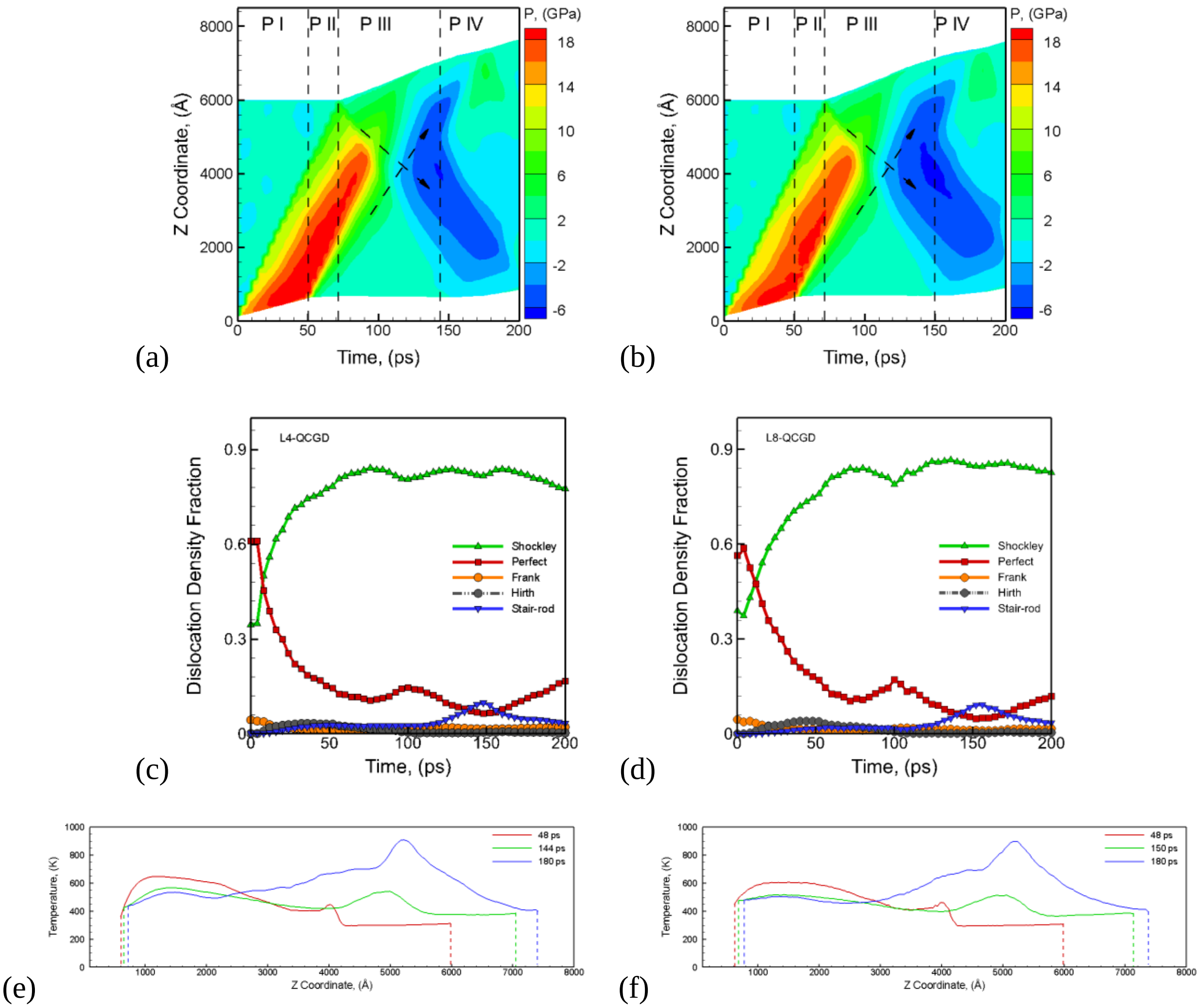

Figure S6: The plots for the evolution of pressure as a function of time during spall failure of 200 nm grain sized polycrystalline Al for a velocity of 1 km/s for the piston as predicted by the (a) L4-QCGD and (b) L8-QCGD simulation. A comparison of the fractions for the different types of dislocations as a function of time is shown in (c) and (d), and corresponding evolution of the temperature at various times for the two simulations is shown in (e) and (f).

The capability of the L4-QCGD simulation is then used to validate the capability of the L8-QCGD simulation to model the shock response of polycrystalline Al system with the same grain orientation relationships. The polycrystalline system chosen corresponds to a grain size of 200 nm with dimensions of 400 nm x 400 nm x 600 nm. This system (atomistic system of ~5.7 Billion atoms) is represented by ~89 Million R-atoms using L4-scaling and ~11 Million R-atoms using L8-scaling in the QCGD simulations. A time-step of 0.020 ps is used for both the simulations. A comparison of the predicted temporal evolution of pressure in the system is shown in Figure S6 (a) and (b), respectively for a piston velocity of 1 km/s and a pulse of 50 ps and is used to compute the spall strength and strain rate values. The L8-scaling retains the shock wave velocities and the computed value of the spall strength of 6.379 GPa with L8-QCGD simulation is slightly higher than the value of 6.062 GPa using L4-QCGD simulations. Similarly,

the comparison plots of the dislocation fractions are shown in Figure S6(c) and (d). The L8-QCGD simulations reproduce the L4-QCGD predicted characteristics related to the nucleation and evolution of dislocation fractions. The plastic deformation behavior results in heating of the metal as shown by the temperature profiles for the two simulations at the end of shock pulse, at peak tensile pressure and after initiation of failure in Figure S6 (e) and (f). These results validate the capability of the L8-QCGD simulations to model the shock response of the polycrystalline system for a grain size of 200 nm and larger and retain the atomistic nature of the nucleation, evolution and interaction of dislocations.

S8. Validation of Scaling Relationships for L16-QCGD Shock Simulations

The L8-QCGD simulations are then used to validate the shock response of L16-QCGD simulations for a 400 nm grain sized polycrystalline system with the same grain orientation relationships. This system, with dimensions of 800 nm x 800 nm x 1200 nm (atomistic system of ~45.6 Billion atoms) is represented by ~89 Million R-atoms using L8-scaling and ~11 Million R-

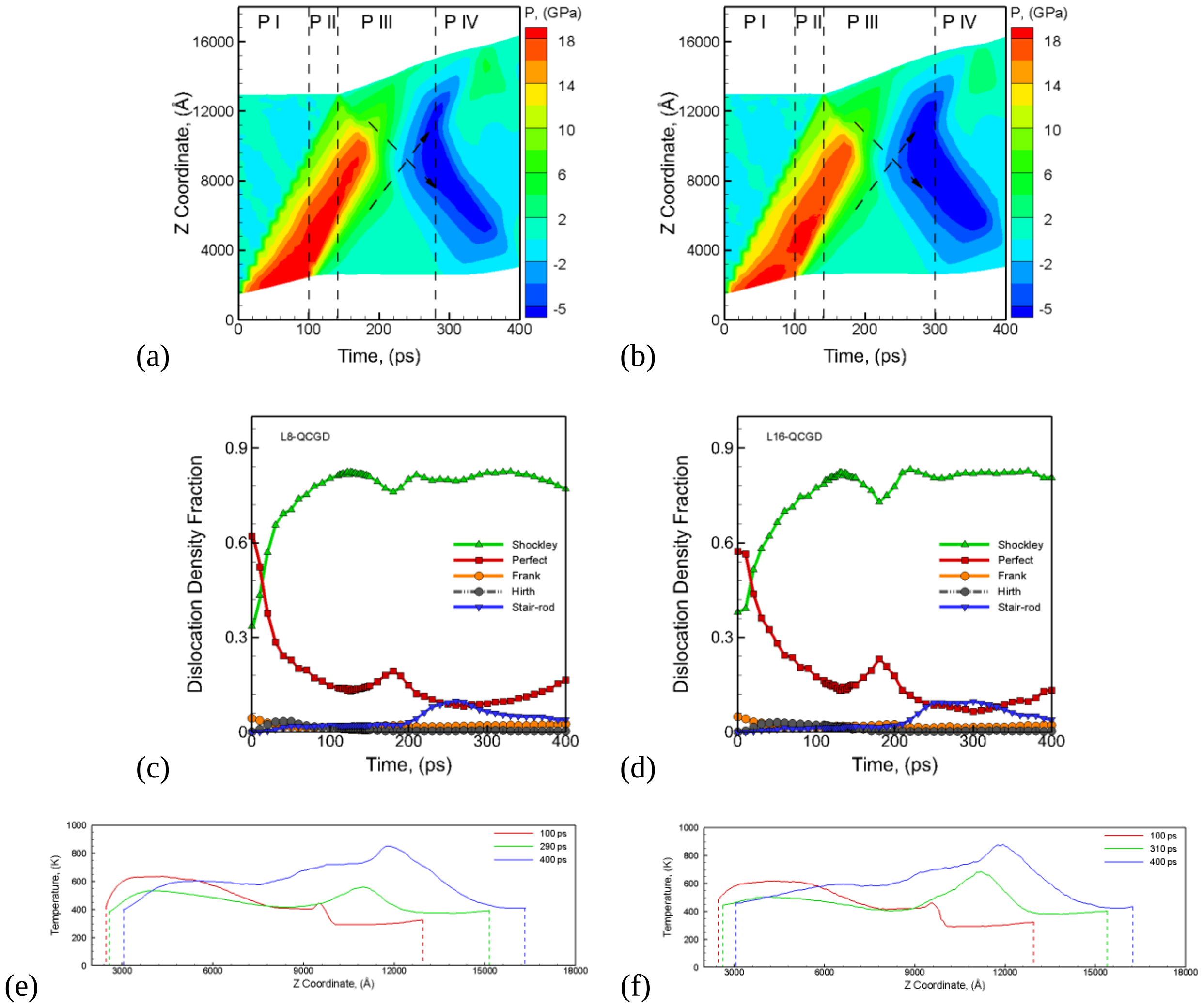

Figure S7: The plots for the evolution of pressure as a function of time during spall failure of 400 nm grain sized polycrystalline Al for a velocity of 1 km/s for the piston as predicted by the (a) L8-QCGD and (b) L16-QCGD simulation. A comparison of the fractions for the different types of dislocations as a function of time is shown in (c) and (d), and corresponding evolution of the temperature at various times for the two simulations is shown in (e) and (f).

atoms using L16-scaling in the QCGD simulations. The comparison of the temporal evolution of the pressure for the two simulations is shown in Figure S7(a) and (b) for a piston velocity of 1 km/s and a pulse of 100 ps. The L16-scaling retains the shock wave velocities and the computed value of the spall strength of 5.989 GPa with L16-QCGD simulation is slightly higher than the value of 5.786 GPa using L8-QCGD simulations. The corresponding evolution of dislocation fractions is shown in Figure S7(c) and (d). The L16-QCGD simulations reproduce the L8-QCGD predicted characteristics related to the nucleation and evolution of dislocation fractions. The comparison of the predicted temperatures at the end of shock pulse, at peak tensile pressure and after initiation of failure for the two simulations is shown in Figure S7(e) and (f). These results validate the capability of the L16-QCGD simulations to model the shock response of the polycrystalline system for a grain size of 400 nm and larger and retain the atomistic nature of the nucleation, evolution and interaction of dislocations.

S9. QCGD Predicted Void Nucleation and Width of Spall

The QCGD simulation used for validation of the various levels of coarsening use same grain orientation relationships for all the polycrystalline Al systems considered here in S4 to S8. The pulse durations used for the various shock loading conditions scale with the system size and are 12.5 ps for MD simulation for system of 150 nm length and grain size of 50 nm, 25 ps for L2-QCGD simulation for system of 300 nm length and grain size of 100 nm, 50 ps for L4-QCGD simulation for system of 600 nm length and grain size of 200 nm, 100 ps for L8-QCGD simulation for system of 1.2 µm length and grain size of 400 nm, and 200 ps for L16-QCGD simulation for system of 2.4 µm length and grain size of 800 nm. For these loading conditions and system sizes, the width of the shock pulse is observed to scale with the level of coarsening and hence the grain size of the system. As a result, the same grain boundaries and triple junctions are observed to experience the triaxial tensile state of stress due to the interaction of the reflected wave with the tail of the compressive wave. As a result, the voids are observed to nucleate at the same grain boundaries and triple junctions for all the systems as these are the weak regions in the polycrystalline metal. The variations in the system sizes and the pulse durations, however, result in variations in the strain rates of deformation at the spall plane and hence result in the variations in the rate of growth of the voids. As a result, the role of microstructure on mechanisms of void nucleation is not investigated here. The variations in the system size, pulse duration and grain size of the metal render variations in the strain rates of loading and hence result in the variations in the nucleation and evolution of voids. The snapshots generated are analyzed to compute the void volume fraction (V_f) in the system and the evolution of the number of voids as a function of time. The analysis of voids suggests that the number of voids reaches a peak value under the triaxial tensile stresses, after which, further growth of the void fraction is only due to the growth of individual voids. As a result, two stages are observed: nucleation of voids followed by growth and coalescence of voids. The snapshots showing the voids in the microstructures of the various simulations discussed in S4 to S8 at a time corresponding to the end of the nucleation stage of the evolution of the void fraction during spall failure are shown in Figure S8. The evolution of void fraction as a function of time is also reproduced by the QCGD simulations. This computed value of the rate of evolution of void fraction is tabulated in Table 2. In addition, the distribution of voids (coordinates in the loading directions) in the system can be used to compute the width of the spall in the various comparison simulations. The calculated values for the width of the spall region generated in all the simulations discussed above at a void fraction of V_f = 0.08 are tabulated in Table S5 for the various levels of coarsening. It can be seen from the comparative

snapshots that the higher level of coarsening retains the void nucleation characteristics predicted by the lower level of coarsening i.e. L2-QCGD/MD, L4-QCGD/L2-QCGD, L8-QCGD/L4-QCGD and L16-QCGD/L8-QCGD.

Table S5: Calculated values for spall strength and width of the spall plane for all the simulations at a void fraction of $V_f = 0.08$ for the various polycrystalline Al systems.

Grain Size (nm)	System Size (nm x nm x nm)	Shock Pulse (ps)	Width of Spall Plane (nm)
50 (MD)	100 x 100 x150	12.5	109.1
50 (L2)	100 x 100 x 150	12.5	100.1
100 (L2)	200 x 200 x 300	25	214.2
100 (L4)	200 x 200 x 300	25	190.5
200 (L4)	400 x 400 x 600	50	417.4
200 (L8)	400 x 400 x600	50	370.8
400 (L8)	800 x 800 x 1200	100	885.6
400 (L16)	800 x 800 x 1200	100	778.3

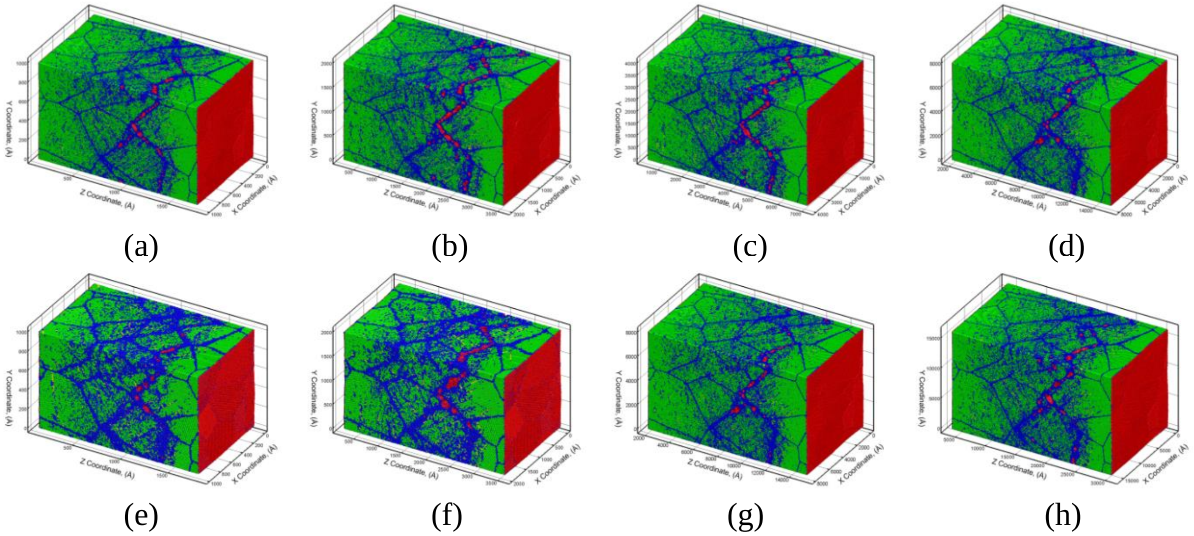

Figure S8: Snapshots showing the nucleation of voids in the polycrystalline Al microstructures of various grain sizes and system sizes at a time corresponding to peak number of voids during spall failure. The snapshots show the nucleation of voids in a 50 nm grain sized system as predicted by (a) MD simulation and (e) L2-QCGD simulation. The snapshots showing the nucleation of voids in a 100 nm grain sized system as predicted by (b) the L2-QCGD simulation and (f) the L4-QCGD simulation. The snapshots showing the nucleation of voids in a 200 nm grain sized system as predicted by (c) the L4-QCGD simulation and (g) the L8-QCGD simulation. The snapshots showing the nucleation of voids in a 400 nm grain sized system as predicted by (d) the L8-QCGD simulation and (h) the L16-QCGD simulation. The coloring of the atoms is used to identify defects, surface and stacking sequences using a combination of CNA and CSP values. The FCC stacked atoms are colored green, HCP stacked atoms are colored yellow, surface atoms are colored red and the disordered atoms are colored blue.

S10. Stair-Rods as Precursors for Void Nucleation

The role of stair-rods in the nucleation of voids is investigated for two simulations using L4-scaling relationships. The first system comprises of a 100 nm grain sized 0.25 µm x 0.25 µm x 0.50 µm system consisting of ~29 Million R-atoms (atomistic system of ~ 1.85 Billion atoms) and the second system comprises of a 200 nm grain sized 0.40 µm x 0.40 µm x 0.50 µm system consisting of ~75 Million R-atoms (atomistic system of ~ 4.8 Billion atoms). The L4-QCGD simulations are carried out for an inward velocity (in the Z direction) of 1 km/s for the piston (bottom end of the system in Z direction) and pulse duration of 50 ps using a time-step of 16 fs. Since the loading conditions and the dimensions of the two systems in the shock direction are same, the spall region width is predicted to be similar for the two systems.

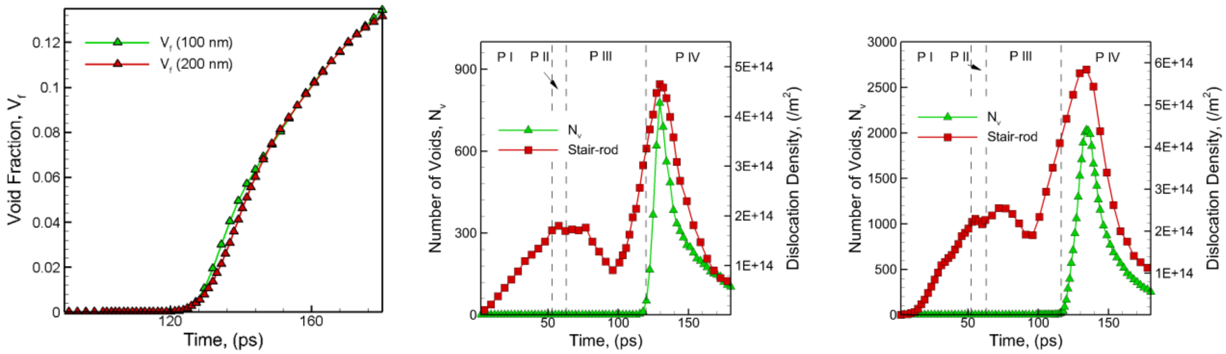

(a) (b) (c)

Figure S9: The plot of the variation of void fraction as a function of time during spall failure of (a) 100 nm and 200 nm grain sized systems as predicted by L4-QCGD simulation for a piston velocity of 1 km/s. A comparison of the evolution of density of Stair-rods and number of voids is shown for (b) 100 nm and (c) 200 nm grain sized systems.

A comparison of evolution of void fraction for the two simulations is shown in Figure S9(a) and suggests that the two systems render a very similar variation. The effect of microstructure is more evident in the evolution of Stair-rods for the two simulations. A comparison of dislocation density of Stair-rods (not converted to atomic scales) and the number of voids as a function of time is shown in Figure S9(b) and (c) for the 100 nm and 200 nm systems, respectively. It can be seen that for both the systems, the density of Stair-rods increases during the nucleation stage of voids (during P-III) and reaches a peak value at the same time as the peak number of voids. This suggests that the high density of Shockley partials in the microstructure that experiences the triaxial tensile stresses interact to form Stair-rods and are likely to form nucleation sites for voids in the spall region. The higher number of grain boundaries for the 100 nm system for the same spall region (width) generates a higher density of such regions at the grain boundaries and hence voids are observed to nucleate at the grain boundaries as shown in Figure 3(e). In comparison, the larger grain interior region for the 200 nm system creates a distribution of Stair-rods at the grain interior in addition to that at the grain boundaries and hence results in void nucleation at the grain boundaries as well as the grain interior regions as shown in Figure 3(f). This correlation between Stair-rods and number of voids is observed in all simulations discussed here.

S11. Scaling Factors for an "Atomistic Spall Strength" value from QCGD simulations

As discussed before, the calculated values for the spall strengths for the various levels of coarsening suggest that the predicted value for QCGD simulations slightly exceeds the values predicted using MD simulations. A comparison of the values suggests that an atomic scaling factor can be defined to predict an atomistic spall strength for these systems modeled using the QCGD simulations. For example, this atomic scaling factor for the spall strength computed using the L2-QCGD simulations is computed as the ratio of the values predicted by MD simulations to that predicted by L2-QCGD simulations as $SCF_{MD/L2}^{SPALL} = \sigma_{MD}^{SPALL} / \sigma_{L2}^{SPALL}$. Similarly, a scaled '*atomistic spall strength*' for the higher levels of coarsening can be computed based on the comparative fractions of the spall strengths. These fractions are obtained using comparisons for the L4-QCGD, L8-QCGD and L16-QCGD systems using the spall strength values for the L2-QCGD, L4-QCGD and L8-QCGD systems, respectively. These fractions are written as:

$$\sigma_{MD/L2}^{SPALL} = \sigma_{MD}^{SPALL} / \sigma_{L2}^{SPALL} =\sim 0.95 \quad (3a)$$

$$\sigma_{L2/L4}^{SPALL} = \sigma_{L2}^{SPALL} / \sigma_{L4}^{SPALL} =\sim 0.95 \quad (3b)$$

$$\sigma_{L4/L8}^{SPALL} = \sigma_{L4}^{SPALL} / \sigma_{L8}^{SPALL} =\sim 0.95 \quad (3c)$$

$$\sigma_{L8/L16}^{SPALL} = \sigma_{L8}^{SPALL} / \sigma_{L16}^{SPALL} =\sim 0.95 \quad (3d)$$

These scaling factors can be used to compute the "*atomistic spall strength*" for the various levels of coarsening as

$$\sigma_{L2}^{At-Spall} = \sigma_{L2}^{SPALL} \cdot \sigma_{MD/L2}^{SPALL} \quad (4a)$$

$$\sigma_{L4}^{At-Spall} = \sigma_{L4}^{SPALL} \cdot \sigma_{MD/L2}^{SPALL} \cdot \sigma_{L2/L4}^{SPALL} \quad (4b)$$

$$\sigma_{L8}^{At-Spall} = \sigma_{L8}^{SPALL} \cdot \sigma_{MD/L2}^{SPALL} \cdot \sigma_{L2/L4}^{SPALL} \cdot \sigma_{L4/L8}^{SPALL} \quad (4c)$$

$$\sigma_{L16}^{At-Spall} = \sigma_{L16}^{SPALL} \cdot \sigma_{MD/L2}^{SPALL} \cdot \sigma_{L2/L4}^{SPALL} \cdot \sigma_{L4/L8}^{SPALL} \cdot \sigma_{L8/L16}^{SPALL} \quad (4d)$$

To test the validity of the atomic scaling factor for spall strength for the various levels of coarsening, the spall strength values are investigated for a different 100 nm grain sized polycrystalline Al system using MD simulations using LAMMPS [48] and L4-QCGD simulations for the same grain orientation relationships. It should be noted that the grain orientation relationships used here are different from the ones used in Section III. The system comprises of dimensions of 0.250 µm x 0.25 µm x 0.50 µm and corresponds to a size of ~1.85 Billion atoms for the MD simulation and ~29 Million R-atoms using L4-scaling for the EAM Al potential [39]. The L4-QCGD simulation was carried out using a timestep of 16 fs. A comparison of the predicted temporal evolution of pressure in the system is shown in Figure S10 (a) and (b), respectively for a piston velocity of 1 km/s and a pulse of 53 ps. The predicted spall strength value for the L4-QCGD simulation of 6.02 GPa is slightly over-predicted as observed for the MD predicted value of 5.50 GPa. The predicted "*atomistic spall strength*" using the scaling relationships discussed above for the L4-QCGD simulation corresponds to a value of $\sigma_{L4}^{At-Spall} = \sigma_{L4}^{SPALL} \cdot SCF_{MD/L4}^{SPALL} =$ 5.44 GPa which is in excellent agreement with the MD-predicted value of 5.50 GPa. Thus, the scaling factors can be used to quantify the "atomistic spall strength" value based on the computed values for the spall strength for any level of coarsening used in the

QCGD simulations and can be used to identify damage tolerant microstructures that span dimensions of several microns and are not accessible using current computing resources to MD simulations.

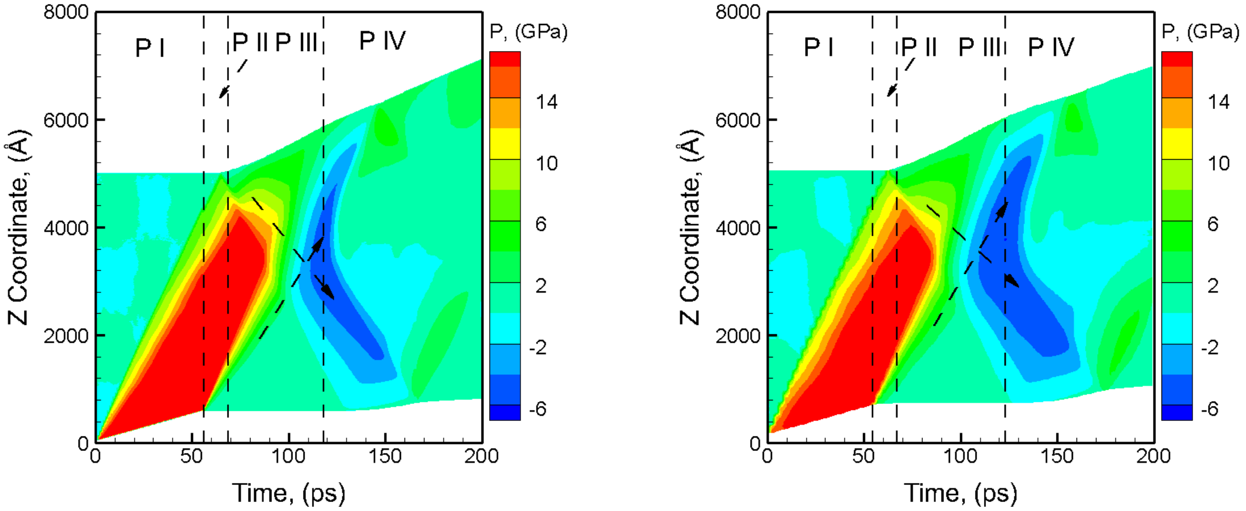

(a) (b)

Figure S10: The plots for the evolution of pressure as a function of time during spall failure of 100 nm grain sized polycrystalline Al for a velocity of 1 km/s for the piston as predicted by the (a) MD and (b) L4-QCGD simulation. The system comprises of dimensions of 0.250 µm x 0.25 µm x 0.50 µm and corresponds to a size of ~1.85 Billion atoms for the MD simulation and ~29 Million R-atoms using L4-scaling.
